# Controlling Nanoscale Compositional Inhomogeneities to Enhance Tb^3+^ Luminescence in YVO_4_ Nanocrystals

**DOI:** 10.1021/acs.inorgchem.6c03156

**Published:** 2026-08-18

**Authors:** Rafael V. Perrella, Ileana Florea, Eric Larquet, Marc Walker, Richard I. Walton, Thierry Gacoin, Paulo Cesar de Sousa Filho

**Affiliations:** † Department of Inorganic Chemistry, Institute of Chemistry, Universidade Estadual de Campinas (Unicamp), R. Monteiro Lobato, 270, 13083-970 Campinas, São Paulo, Brazil; ‡ Laboratoire de Physique de la Matière Condensée, Ecole Polytechnique, Institut Polytechnique de Paris, CNRS, Route de Saclay, 91128 Palaiseau, France; § Laboratory of Physics of Interfaces and Thin Films, Ecole Polytechnique, Institut Polytechnique de Paris, CNRS, 91228 Palaiseau, France; ∥ Université Cote d’Azur (UniCA), CNRS, CRHEA, 06905 Sophia Antipolis Cedex, France; ⊥ Synchrotron SOLEIL, 91192 Gif-sur-Yvette, France; # Department of Physics, 2707 University of Warwick, Coventry CV4 7AL, United Kingdom; ∇ Department of Chemistry, University of Warwick, Gibbet Hill Road, Coventry CV4 7AL, United Kingdom

## Abstract

Controlling chemical homogeneity during aqueous coprecipitation of oxide nanoparticles remains challenging due to unintended byproducts that impact the microstructure and functionality of the final materials. Hence, precise tuning of structural, optical, and catalytical properties requires not only accurate knowledge of how such impurities evolve under annealing, but also effective strategies for defect engineering. Here, we demonstrate how hydroxylation and carbonation affect the internal structure and optical properties of YVO_4_ nanoparticles synthesized using different vanadate precursors. The use of highly basic Na_3_VO_4_ promotes unintentional carbonation, leading to Y/V nonstoichiometry and formation of the yttrium-rich Y_8_V_2_O_17_ secondary phase upon annealing. Combined *in situ* and *ex situ* characterizations further reveal that CO_3_^2–^ and OH^–^ species embedded in the particle structure induce thermally driven nanovoids and reshape the local defect structure of the solids. More importantly, we expand defect engineering in YVO_4_:Tb^3+^ nanoparticles by intentional hydroxylation and carbonation to enhance luminescence intensities. Adjusting these compositional defects enables us to modulate the energy of metal–metal charge transfer (MMCT) deactivation pathways, resulting in up to 13-fold higher emission intensity compared to chemically pure YVO_4_:Tb^3+^. These findings establish defect incorporation as an effective route for tailoring the optical response of rare-earth vanadate nanoparticles, opening new opportunities for advanced photonic and luminescent sensing applications.

## Graphical abstract

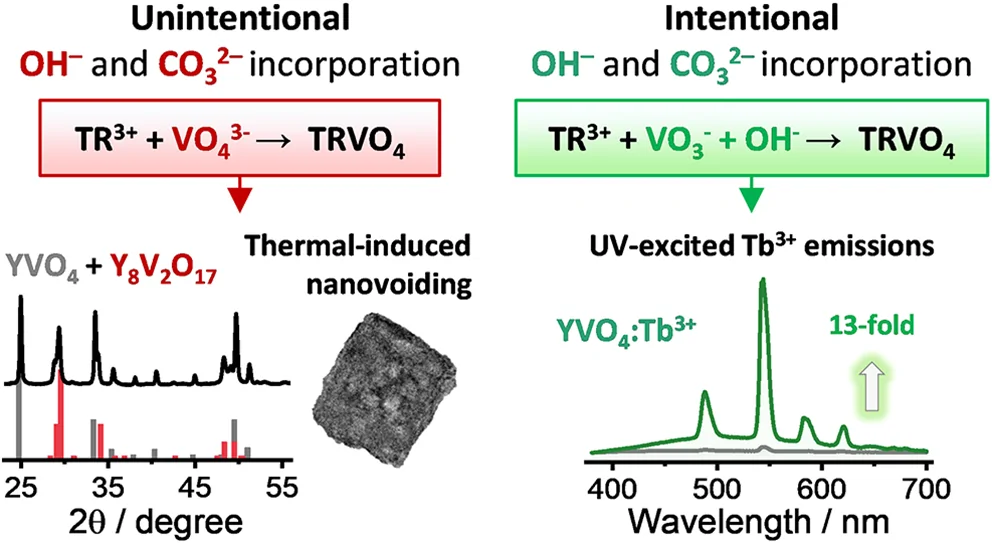

## Introduction

The synthesis of nanoparticles is a field of intense research driven by the pursuit of precise control over key parameters that define their properties, such as composition, crystallinity, size, and morphology.^1,2^ While achieving this level of control involves careful selection of precursors and fine-tuning of experimental conditions to master the mechanisms that govern particle formation, emerging applications in optics and catalysis require further manipulation of the internal microstructure and defect chemistry of the nanoparticles.^3,4^ This challenge applies for rare-earth orthovanadate solids (REVO_4_), which are remarkably versatile host lattices for light-emitting applications.^5–7^ REVO_4_ nanoparticles enable multiple emissions from lanthanoid (Ln^3+^) dopants under ultraviolet (UV) or near-infrared (NIR) excitation, thus taking advantage of both downshift and upconversion processes.^8,9^ Recent advances evidence the potential of REVO_4_ in emerging fields such as nanothermometry,^9,10^ bioimaging,^11^ and chemical sensing,^12^ including detection and generation of reactive oxygen species (ROS).^13,14^ Additionally, REVO_4_ solids show a complex defect chemistry due to the redox activity of vanadium (V^3+^/V^4+^/V^5+^) and some of the lanthanides (Ln^2+^/Ln^3+^/Ln^4+^) species combined to oxygen vacancies (*v*_O_^••^).^14–16^ The concentration of these defects in REVO_4_ nanoparticles is influenced by an intricate interplay between synthesis methodology, surface state, and composition, enabling interesting catalytic properties owing to oxygen mobility, redox activity, and Lewis/Brønsted acidity.^3,14,16^

The large interest in REVO_4_ materials also stems from their ability to crystallize at low temperatures, which enables synthetic procedures such as hydrothermal and coprecipitation methods.^17,18^ Despite its apparent simplicity, however, coprecipitation often provides nanoparticles with rather undefined microstructures, because the formation of REVO_4_ solids from aqueous precursors follows nonclassical precipitation pathways.^19,20^ In aqueous precipitations, REVO_4_ nanoparticles are typically formed by aggregation of kinetically stable intermediate noncrystalline phases, followed by crystallization of the tetragonal vanadate phase.^21,22^ The precipitation kinetics and the occlusion of hydroxides and carbonates can culminate in deviations in the RE/V ratio in the final REVO_4_ particles, which significantly impacts their electronic structure. For instance, our recent investigation showed how chemical inhomogeneities affect the optical properties of nanostructured YVO_4_:Tb^3+^ solids.^4^ Even though deviations from expected compositions contribute to the luminescence mechanism of Tb^3+^ in YVO_4_, which was classically considered a nonluminescent material, it remains challenging to control accurately the carbonate and hydroxide content in the final solids.

In addition to electronic effects, the internal microstructure and chemical homogeneity also directly impact on how the nanoparticles evolve upon annealing, which is crucial for controlling phase purity and for the accurate tailoring of the materials for optical and catalytic applications.^23–25^ A comprehensive view of the dynamics of physicochemical processes taking place on thermal treatment of nanoparticles requires combining direct observation by *in situ* heating transmission electron microscopy (TEM) and *ex situ* analysis provided by conventional structural characterization.^23,24^ These techniques have been applied to multiple oxides,^23–25^ providing in-depth understanding of solid-state mechanisms and affording insights for the design of nanoparticles. In this work, we provide an example of microstructural engineering of oxide nanoparticles, showing the connections between composition, structure and functionality of rare-earth vanadates. We demonstrated the effects of unintentional contaminations present in YVO_4_ nanoparticles, probing the thermal induced nanovoiding process in these solids arising from the decomposition of carbonate and hydroxide impurities. Furthermore, we expanded our previous observations concerning Tb^3+^ luminescence in YVO_4_ by analyzing systematically the effects of carbonation and hydroxylation, comparing coprecipitation procedures using Na_3_VO_4_ or NaVO_3_ as vanadate precursors. Intentional defect engineering in YVO_4_ nanoparticles by modification with CO_3_^2–^ and OH^–^ using NaVO_3_ as precursor enables us to modulate the intrinsic nonradiative deactivation routes of the ^5^D_4_ emitting state, providing enhanced Tb^3+^ emissions. These results open new perspectives for selective activation/deactivation of Tb^3+^ luminescence for sensing purposes.

## Experimental Section

### Chemicals

Rare-earth oxides (Y_2_O_3_ and Tb_4_O_7_, 99.99%), sodium orthovanadate (Na_3_VO_4_, 99.98%), sodium metavanadate (NaVO_3_, >98%), and sodium carbonate (Na_2_CO_3_, 99.5%) were used as received from Sigma-Aldrich. YCl_3_ and TbCl_3_ solutions (0.1 mol L^–1^) were prepared by dissolution of the previously calcined oxides in concentrated HCl (37% m/m, Synth, analytical grade) at 70 °C. A small amount of hydroxylamine hydrochloride (NH_2_OH.HCl, 99%, Sigma-Aldrich) was added to ensure total reduction of Tb^4+^. The final pH of these solutions was regulated to 5 by solvent evaporation. A 1 mol L^–1^ of NaOH (97%, Synth) aqueous solution was also prepared and stored at 4 °C for further use.

### Synthesis

Rare-earth vanadate nanoparticles were prepared at 25 °C via colloidal coprecipitation^18,26,27^ using either orthovanadate (Na_3_VO_4_) or metavanadate (NaVO_3_) as precursors. In a typical synthesis of YVO_4_ nanoparticles with Na_3_VO_4_, an YCl_3_ solution (0.1 mol L^–1^) was added dropwise into a freshly prepared orthovanadate aqueous solution (0.1 mol L^–1^, pH = 12.5–13) of the same volume under vigorous stirring. Few drops of 1 mol L^–1^ NaOH were added to maintain the pH between 12–12.5. The suspension was stirred for 30 min and purified by dialysis against Milli-Q water until its conductivity dropped below 100 μS cm^–1^. The solids were recovered by centrifugation at 26300*g* for 30 min. The total amount of powder nanoparticles (*ca*. 800 mg) was divided into seven portions. One portion was kept at room temperature (hereafter named as-synthesized sample), while the remaining six portions were placed into separate porcelain crucibles and heat treated under either in air or nitrogen (N_2_) atmospheres. The solids were heated at 100, 300, 500, 700, 900, and 1100 °C using a heating rate of 10 °C min^–1^. Once each target temperature was reached, the samples were held at that temperature for 10 min. For comparative purposes, YVO_4_ particles were prepared using a two-year-old open flask of Na_3_VO_4_ and heat-treated at the same temperatures under N_2_ atmosphere.

For the synthesis using NaVO_3_, 50 mL of an aqueous NaVO_3_ solution (0.1 mol L^–1^) was prepared, supplemented with NaOH (2 or 2.5 equiv with respect to VO_3_^–^, from the 1 mol L^–1^ stock solution) and Na_2_CO_3_ (0 or 0.5 equiv relative to VO_3_^–^). Subsequently, 50 mL of an aqueous YCl_3_ solution (0.1 mol L^–1^) was rapidly added under vigorous stirring. After 30 min, the colloidal suspensions were purified by dialysis against Milli-Q water for 24 h. The solid particles were recovered by centrifugation at 26300*g* for 30 min, followed by redispersion in 40 mL of acetone and additional centrifugation. Samples were designated as YV2, YV2.5, YV2C0.5, and YV2.5C0.5, where YV*n* and YV*n*C*x* indicate syntheses performed with *n* equivalents of NaOH and *x* equivalents of Na_2_CO_3_ relative to the metavanadate precursor. The synthesis of Tb-doped YVO_4_ particles followed a similar procedure, using appropriate mixtures of rare-earth chloride solutions (YCl_3_ and TbCl_3_, 95% Y^3+^, 5% Tb^3+^, mol/mol).

### Characterization

Powder X-ray diffraction (XRD) measurements were carried out on a Bruker D8 Advance diffractometer equipped with a Cu tube (Kα_1_ and Kα_2_ wavelengths), scanning the 10–80° 2θ range with an increment of 0.02°. Lattice parameters, cell volume, coherence lengths, and microstrains were determined by Rietveld analysis using MAUD software.^28^ The infrared (ATR-FTIR) spectra were acquired on an Agilent Cary 630 ATR spectrometer on a diamond crystal, with a 4 cm^–1^ resolution. Thermogravimetric analysis (TGA) was performed simultaneously with mass spectrometry using a Mettler Toledo TGA/DSC 1–600 instrument coupled to a Hiden HPR-20 QIC R&D system and a triple-filter mass spectrometer with SEM detection. The sample was heated up to 900 °C at 10 °C min^–1^, using N_2_ as a carrier gas to minimize background water and to eliminate CO_2_ from the background. X-ray photoelectron spectroscopy (XPS) measurements were acquired on a Kratos AXIS Ultra DLD instrument using an ultrahigh vacuum system with a base pressure of 5 × 10^–11^ mbar. The samples were excited with X-rays from a monochromatic Al Kα source (*h*υ = 1,486.7 eV), with the photoelectron being detected at a 90° takeoff angle. Peak fitting was carried out using the CasaXPS package,^29^ incorporating Voigt (mixed Gaussian–Lorentzian) line shapes and a Shirley background. Real-time *in situ* heating transmission electron microscopy (TEM) and selected area electron diffraction (SAED) were performed using an environmental transmission electron microscope (Titan ETEM “NanoMAX”) at 300 kV, equipped with a Cs image corrector, an UltraScan 2k × 2k CCD camera, and a direct electron K2 camera. All observations were conducted using a Protochips Fusion sample holder. We used specific heating membranes with silicon nitride windows provided with the sample holder. Temperature control was managed by the sample holder, with calibration provided by the manufacturer. Prior to observation, 5 μL of particle suspension (∼0.1 g L^–1^) was deposited on the surface of the silicon nitride window and left to dry. The sample was heated at 10 °C min^–1^ from 25 to 900 °C, with 15–30 min stabilization periods at each step, while different regions were imaged in bright-field mode. Diffuse reflectance spectra of powder particles were acquired in the 200–800 nm range on a Shimadzu 2550 spectrometer equipped with deuterium and tungsten-halogen lamps, using barium sulfate as blank. The reflectance measurements were mathematically converted into absorbance using the Kubelka–Munk transformation via the apparatus software. Luminescence spectra were recorded on a Fluorolog 3 (Horiba FL3–22-iHR320) spectrofluorometer equipped with a Hamamatsu R928P photomultiplier detector and a 450 W xenon arc lamp. Spectra were corrected according to the lamp intensity, excitation and emission optical systems, and photomultiplier response via the software apparatus. Excited state decay curves were obtained on the same instrument using a TCSPC system and a 150 W xenon pulsed lamp as the excitation source. Suspensions in H_2_O and D_2_O were prepared by drying 10 mg of powder samples and resuspending the particles in 1 mL of H_2_O or D_2_O in an ultrasound bath for 60 min.

## Results and Discussion

The preparation of REVO_4_ aqueous colloids is usually undertaken in alkaline conditions to ensure high concentration of free H_*x*_VO_4_^3‑*x*^ species with respect to polyvanadates (*i.e*. (VO_3_)*_n_* ^*n*–‑^).^18,30^ The steps involved in the precipitation of the REVO_4_ nanoparticles are complex and may comprise amorphous-to-crystalline conversions via kinetically stable intermediates,^21,22,26^ ultimately defining crucial parameters such as particle sizes, morphologies, and internal crystallinity. However, these parameters are also affected by the choice of the vanadate precursor, which also plays an important role in the unintentional introduction of minor secondary components in the final nanoparticles, such as carbonates and hydroxides.^26^ Such contaminants may go unnoticed by conventional characterization techniques, but the expected functionality of pristine particles may be enhanced or fully suppressed by such compositional variations. In turn, the presence of carbonates and hydroxides embedded in the REVO_4_ lattice also affects the RE/V ratio, which can culminate in unexpected phases after elimination of CO_2_ and H_2_O by annealing.

For instance, the use of sodium orthovanadate as a vanadate precursor in pHs exceeding 12 ^9,11,18^ often results in undesired compositional variability due to the high tendency of Na_3_VO_4_ powder and its aqueous solutions to trap atmospheric CO_2_. The pristine nanocrystalline YVO_4_ particles obtained by this well-known aqueous coprecipitation with Na_3_VO_4_ show an apparently pure tetragonal phase (Figure 1a). However, amorphous residual hydroxide and carbonate domains occur in these particles [FTIR (Figure 1b): 3300 cm^–1^ (υOH); 1640 cm^–1^ (δHOH); 1508 + 1390 cm^–1^ (υCO_3_^2–^); 900–650 cm^–1^ (υ_3_VO_4_^3–^)], which results in an unexpected behavior upon annealing. While higher temperatures result in a decrease of these bands due to the elimination of OH^–^ and CO_3_^2–^ (Figure 1b), XRD shows the formation of a phase around 700 °C, identified as the tetragonal Y_8_V_2_O_17_ structure (JCPDS 44–0390,^31^ Figure 1a). The crystallization of this yttrium-rich phase upon annealing occurred both in air and N_2_ atmosphere (Figure S1) suggesting a negligible effect of an oxidizing or inert environment on this process. This phase remained stable for 12 h at 1000 °C (Figure S2), which indicates a high thermodynamic stability.

**Figure 1:**
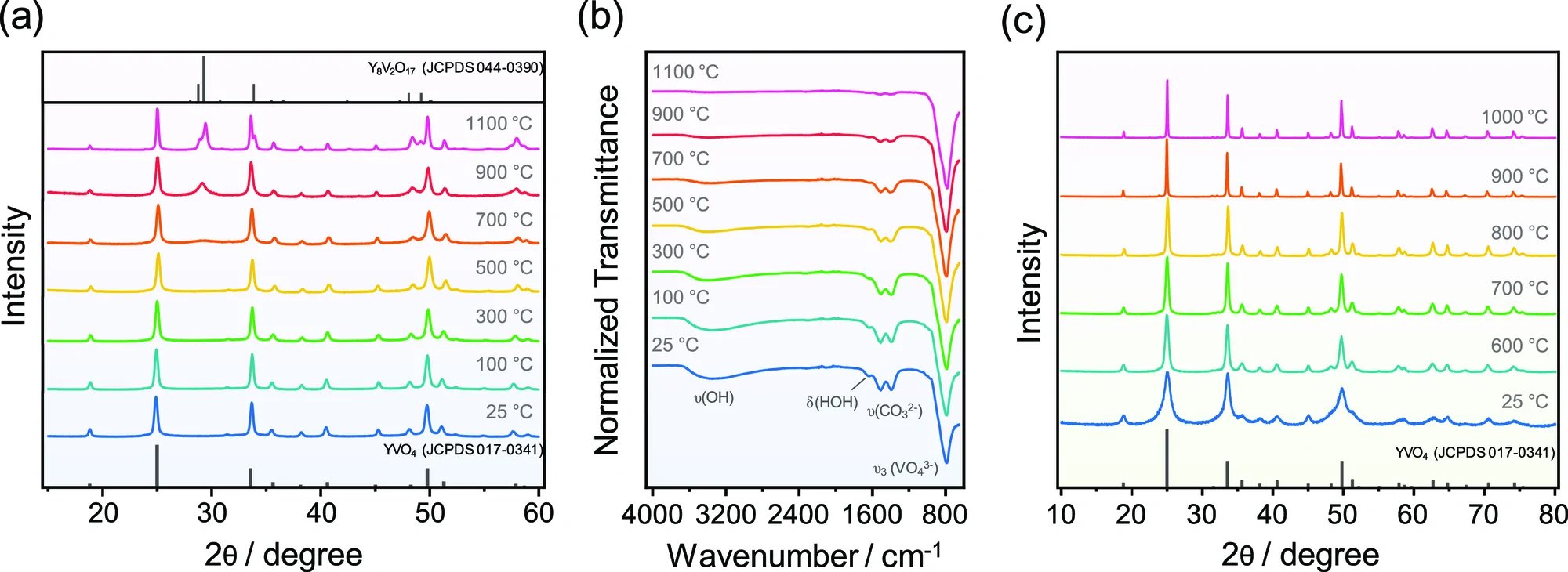
(a) Powder XRD patterns and (b) FTIR spectra of YVO_4_ nanoparticles synthesized at 25 °C using Na_3_VO_4_ precursor and annealed in air between 100 and 1100 °C. (c) Powder XRD patterns of YVO_4_ nanoparticles synthesized at 25 °C using NaVO_3_ precursor with two equivalents of NaOH and annealed between 600 and 1000 °C.

These results underscore a poor compositional control caused by the use of highly basic Na_3_VO_4_ in the synthesis of the REVO_4_ nanoparticles. For comparison, REVO_4_ solids can also be synthesized with significantly higher compositional purity using sodium metavanadate (NaVO_3_) as the vanadate precursor.^26^ In contrast to Na_3_VO_4_, sodium metavanadate is composed of the less basic (VO_3_)_n_^n‑^ chains, and it is significantly less prone to carbonation. In this case, metavanadate chains are converted *in situ* into orthovanadate ions by addition of at least two equivalents of OH^–^ (*i.e*., VO_3_^–^ + 2OH^–^ → VO_4_^3–^ + H_2_O), where addition of precisely controlled amounts of hydroxide ions avoids unbalanced Y/V stoichiometries from the unintentional presence of CO_3_^2–^ and OH^–^ in the REVO_4_ nanoparticles as kinetic products. Consequently, thermal treatment at 1000 °C of YVO_4_ particles synthesized from NaVO_3_ precursor supplemented with two equivalents of NaOH resulted in structurally pure *I*4_1_/*amd* tetragonal solids showing no secondary phases (Figure 1c).

Hence, even though particles prepared by Na_3_VO_4_ and NaVO_3_ precursors can both be labeled as “YVO_4_”, deviations in the intrinsic Y/V stoichiometry result in distinctly different behavior upon annealing, where vanadium deficiency due to OH^–^ and CO_3_^2–^ impurities causes the appearance of secondary crystalline phases. This is further highlighted by comparing YVO_4_ particles prepared by Na_3_VO_4_ precursors of different ages (Figure 1 and Figures S3 and S4). While particles described in Figure 1a,b were obtained using a newly opened sodium orthovanadate container, the use of a two-year-old opened, highly carbonated Na_3_VO_4_ resulted in particles exhibiting significantly more OH^–^ and CO_3_^2–^ groups. This is confirmed by measuring the intensity ratio between the carbonate (ν­(CO_3_^2–^)) and vanadate (ν_3_(VO_4_^3–^)) asymmetric stretching bands in IR spectra (Figure S4c). Such a higher vanadium deficiency due to OH^–^ and CO_3_^2–^ groups present in the aged Na_3_VO_4_ precursor results in obvious effects after thermal treatment (Figure S3). Most evidently, there is a decrease in the temperature required for the initial crystallization of the Y_8_V_2_O_17_ secondary phase (from ∼700 °C to ∼500 °C), as indicated by the intensity of the peak at 2θ∼29° in XRD patterns. Furthermore, the amount of Y_8_V_2_O_17_ formed at 1100 °C is clearly higher when the old precursor was used (Figures 1 and S3), thus indicating that compositional deviations in the final YVO_4_ particles depend directly on the carbonation degree of the vanadate precursor.

The formation of the yttrium-rich phase (Y_8_V_2_O_17_) upon thermal treatment of YVO_4_ nanoparticles is a consequence of Y/V unbalance in pristine particles and ultimately highlights how subtle parameters may affect the properties of the final solids. To further investigate this, we demonstrate that the processes involved in the thermal decomposition of pristine YVO_4_ particles containing unintentional OH^–^ and CO_3_^2–^ are not restricted to the surface of the solids.

First, it is important to note that formation of the secondary Y_8_V_2_O_17_ phase is not caused by evaporation of vanadium oxides from YVO_4_. As reported elsewhere,^32–37^ YVO_4_ can decompose into yttrium-rich phases releasing V_2_O_5_ but only at temperatures exceeding 1200 °C [*e.g*., 8YV*O*_4_(*s*) → Y_8_V_2_O_17_(s) + 3 V_2_ *O*_5_(*g*), which can also be written as 4Y_2_O_3_.4 V_2_ *O*_5_(*s*) → 4Y_2_O_3_.V_2_ *O*_5_(*s*) + 3 V_2_ *O*_5_(*g*)]. The formation of Y_8_V_2_O_17_ begins in significantly lower temperatures in our case, but we ruled out this possibility by coupled thermogravimetry and mass spectrometry (TG-MS) measurements, which showed that no signals related to vanadium species (*e.g*., V_4_O_10_^+^ and V_4_O_8_^+^) were detected (Figure S5). Instead, the mass loss (Figure S5) accompanying the heat-treatment is attributed only to the release of H_2_O­(g) (*m*/*z* = 18) and C*O*_2_(*g*) (*m*/*z* = 44) from CO_3_^2–^ and OH^–^ contaminants in the YVO_4_.

Based on the above observations, YVO_4_ particles prepared from Na_3_VO_4_ precursors must be considered having an actual composition Y­(VO_4_)_1–0.66x‑0.33y_(CO_3_)_*x*_(OH)_*y*_, where carbonate and hydroxide impurities are embedded in the YVO_4_ structure. X-ray photoelectron spectra (XPS) clearly demonstrate the presence of carbonate species at the surface of as-synthesized particles by observed components at ∼293.4, 292.0, and 290.2 eV (Figure S6). Compositional analysis of XPS spectra further revealed a high vanadium deficiency at the surface with an Y/V molar ratio of 3.5 (Figure S6) caused by the presence of high amounts of carbonates and hydroxides. However, acid leaching at pH = 3 of pristine nanoparticles provides negligible removal of carbonate species (Figure S7), as indicated by the nearly constant ratio between intensities of ν­(OH)+ν­(CO_3_^2–^) stretching bands with respect to the vanadate ν_3_(VO_4_^3–^) band in IR spectra before and after exposition of the particles to an HCl solution (pH = 3). Therefore, carbonate and hydroxide impurities are not limited to the surface and a significant proportion occupy sites within the nanoparticle structure.

The presence of occluded carbonate/hydroxide in the YVO_4_ nanoparticles and their morphological/structural effects on the thermal behavior of these solids was further probed by TEM with *in situ* heating (Figure 2 and S8–S12). While as-obtained particles show low-density, amorphous regions both at the surface and within the inner volume at room temperature, the increase in temperature (400 °C) triggers the elimination of CO_2_ and H_2_O via thermal decomposition of hydroxides [*i.e*., 2OH_O_^•^ → O_O_^x^ + *v*_O_^••^ + H_2_O­(g)] and carbonates [C_V_′ + 2O_O_ *^x^* → 2*v*_O_^••^+*v*_V_'''' + CO_2_(*g*)], both leaving oxygen vacancies (the symbols in the equations refer to the Kröger-Vink notation). As the heating proceeds, the vacancies coalesce into <2 nm voids that are homogeneously distributed throughout the nanoparticles (Figures 3 and S12). Further heating induces progressive coarsening of the high-curvature small voids into pores of 5–10 nm, which is likely driven by reduction of the total interfacial energy analogous to the Ostwald ripening phenomenon.^23–25,38^ The internal porosity evolves until temperatures higher than 750 °C are reached, where the solid undergo densification and intra- and interparticle sintering (Figures 3 and S8, S9, and S12), collapsing the discontinuities inside the particles. Elimination of CO_3_^2–^ between 500 and 1000 °C is also evidenced by the reduction of carbonate peak at high binding energies in the C 1s XPS spectra (Figure S6).

**Figure 2:**
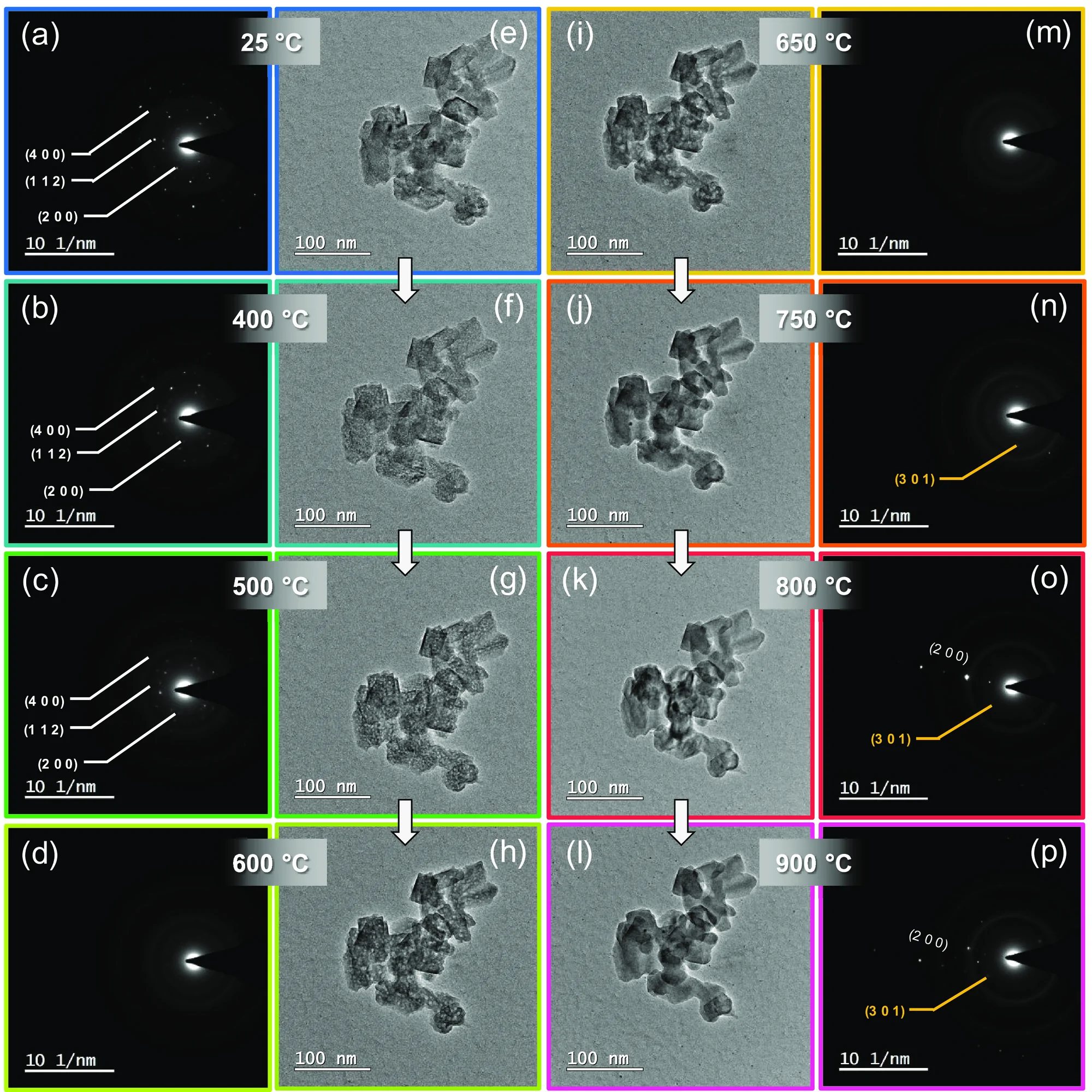
*In situ* TEM with heating (a–d, m–p) SAED and (e–l) bright field images of YVO_4_ nanoparticles synthesized at 25 °C using Na_3_VO_4_ precursor. The characteristic (400), (112), and (200) crystalline planes (white) of the tetragonal YVO_4_ phase and the (301) plane (yellow) of the tetragonal Y_8_V_2_O_17_ phase are indicated in the SAED images.

**Figure 3:**
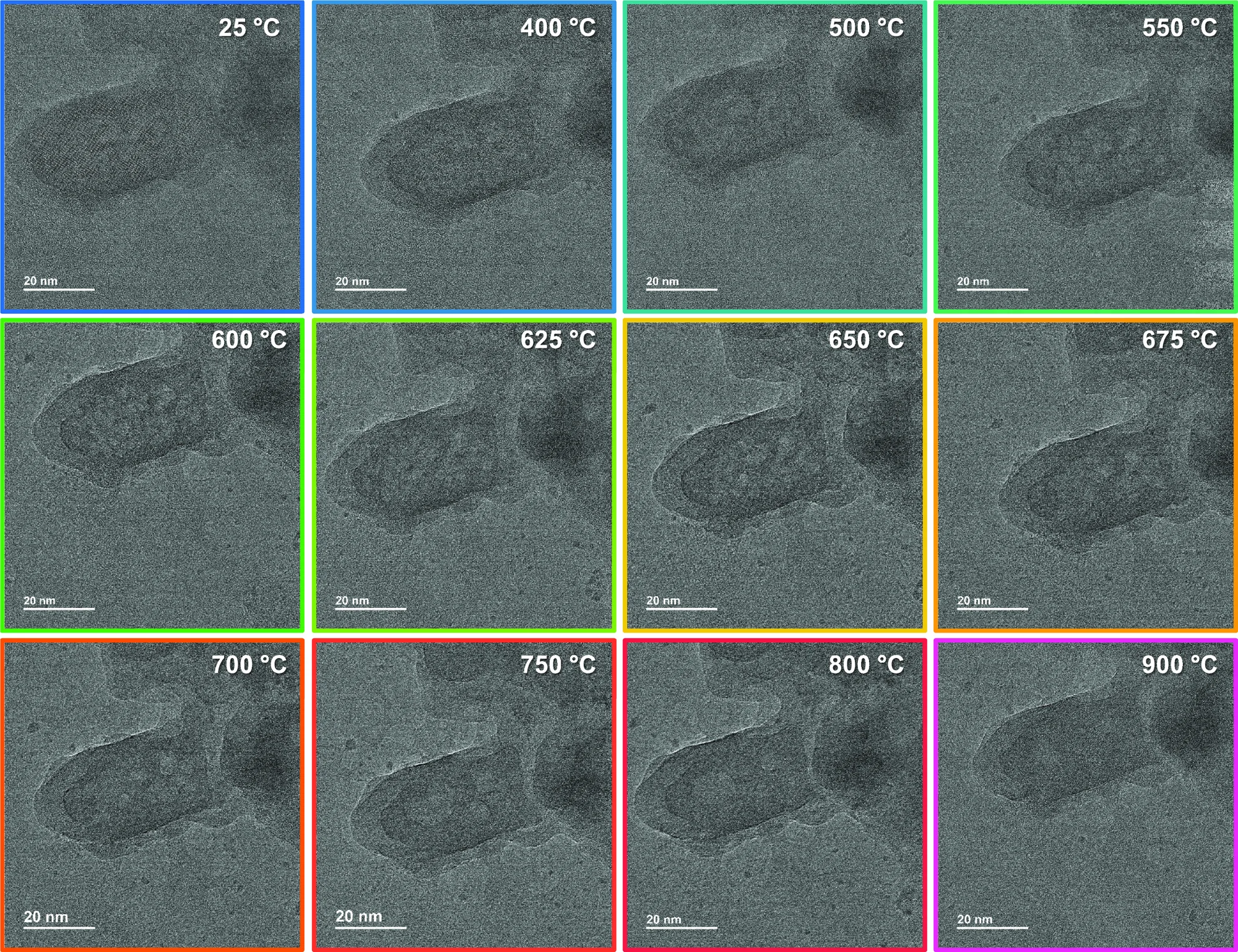
*In situ* high-resolution TEM images upon heating of YVO_4_ nanoparticles synthesized at 25 °C using Na_3_VO_4_ precursor.

Parallel to the nanovoiding process, the alterations in the internal structure of the nanoparticles also involve reorganization of the crystalline domains and evolution of the Y_8_V_2_O_17_ phase. Powder XRD analysis performed with solids annealed at 1 atm in air (Figures 1 and 4 and Table S1) agrees very well with the observations in *in situ* TEM heating in high vacuum. First, while progressive annealing of the nanostructures is expected to provide thermal energy for crystallite coalescence and increase the crystalline alignment, our observations by powder XRD and *in situ* TEM show the opposite behavior. The crystalline coherence lengths calculated from powder XRD (Figure 4c) decrease at temperatures higher than 100 °C, when the nanovoiding process starts and limits the crystalline orientation throughout the nanoparticles. Similarly, selected area electron diffraction (SAED) patterns confirm the polycrystalline nature of the as-synthesized particles (Figures 2 and S10 and S11), where decreasing intensities of the diffraction signals associated with the YVO_4_ crystalline planes between 400 and 650 °C confirm the decrease in the crystalline coherence. Furthermore, the annealing also induces an increase of crystalline microstrain in comparison to as-synthesized solids (Figure 4c), which is also a consequence of numerous high-curvature interfaces inside the nanoparticles. The cell parameters calculated from powder XRD analysis revealed that the effects of the annealing are not homogeneous in the different crystalline directions of the tetragonal structure, with a contraction in the *a*/*b* parameters (*i.e*. [100] and [010] directions) with an expansion in *c* ([001] direction). Consequently, a decrease in the unit cell volumes was observed between 25 and 500 °C (Figure 4a,b), which is primarily caused by the elimination of carbonate and hydroxide defects and densification of the tetragonal phase.^4,27^ This unit cell contraction might also be partially attributed to the evolution of the voids observed by *in situ* TEM in single nanoparticles. For instance, the images demonstrate that the particles keep a nearly constant contour until intra- interparticle sintering takes place at temperatures higher than 750 °C. Hence, considering that the volume of individual nanoparticles is nearly constant, the formation of the nanovoids is also expected to promote a minor contraction of unit cells. At temperatures above 750 °C, the collapse of the internal porosity is accompanied by an increase in coherence lengths, decrease of microstrain and expansion of the unit cells (Figure 4b,c). These processes correlate well with XPS results of samples heated *ex situ* (Figure S6), which suggest an oxygen uptake at high temperatures with oxidation of V^3+^ and V^4+^ defects to V^5+^ and a decrease in the amount of Y^3+^ sites bound to oxygen defects at the surface (Figure S6d,h,l). Finally, formation of Y_8_V_2_O_17_ at temperatures above 750 °C is detectable not only by *ex situ* thermal treatment in air (powder XRD, Figure 1), but also at *in situ* high vacuum conditions, showing the appearance of the (3 0 1) reflection of the Y_8_V_2_O_17_ phase in SAED patterns (Figures 2 and S10 and S11).

**Figure 4:**
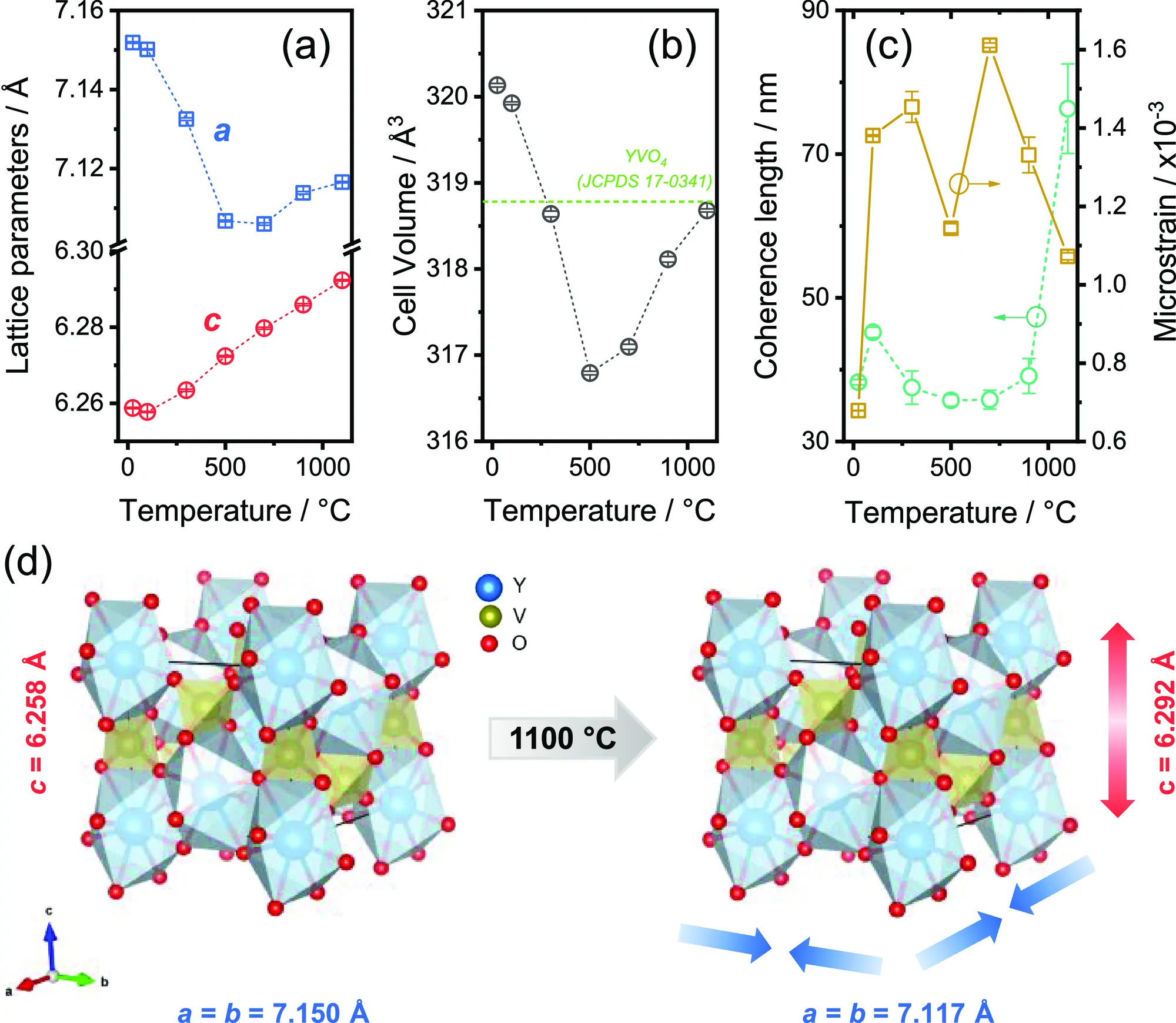
Evolution of the (a) tetragonal lattice parameters (*a,c*), (b) unit cell volume, and (c) coherence length and microstrain with calcination temperature of the YVO_4_ nanoparticles synthesized using Na_3_VO_4_ precursor. (d) Unit cell of tetragonal YVO_4_ (space group *I*4_1_/amd) highlighting the lattice parameter changes after annealing at 1100 °C.

Our results highlight the very good correlation between the *in situ* TEM observations in individual nanoparticles and the bulk, collective *ex situ* XRD analysis. Thus, a clear picture on the effects of thermal decomposition of embedded hydroxide and carbonate species in the YVO_4_ nanoparticles is provided.

Considering this complex thermal behavior introduced by the unintentional impurities in YVO_4_ nanoparticles synthesized using Na_3_VO_4_, the use of metavanadates as precursors enables not only structurally and chemically pure products, but also the possibility that these contaminants might modulate properties of interest. In particular, such a microstructure engineering may modulate luminescent properties. While some optical applications are limited by low quantum yields and require thermal annealing to achieve dense and highly crystalline YVO_4_ nanoparticles with improved emission features,^9,39–42^ other luminescent properties are only activated in the presence of a high concentration of defects. This is the case for the luminescence of Tb^3+^ in YVO_4_ solids, which has been demonstrated to be dramatically dependent on the concentration of defects and lattice distortions.^4^ To demonstrate this point, we produced YVO_4_ nanoparticles intentionally hydroxylated and carbonated via careful regulation of CO_3_^2–^/OH^–^ in the colloidal synthesis using NaVO_3_ as precursor adding Na_2_CO_3_ and NaOH in known amounts, as described in the Experimental section.

Table S2 summarizes the structural properties of investigated compositions, referred to as YV2 (chemically pure YVO_4_, Figure 1), YV2C0.5 (carbonated YVO_4_), YV2.5 (hydroxylated YVO_4_), and YV2.5C0.5 carbonated/hydroxylated YVO_4_, (Figure S13). The YV2 and YV2.5 particles exhibit polycrystalline nature and similar elongated shape, with carbonated particles (YV2C0.5, YV2.5C0.5) showing lower aspect ratio (Figure S14), in agreement with previous reports.^26^ While annealing of the YV2 sample at 1000 °C yields chemically and structurally pure YVO_4_, thermal treatment of the intentionally carbonated/hydroxylated particles with a Y/V molar ratio higher than 1 (YV2C0.5, YV2.5, and YV2.5C0.5) resulted in the formation of yttrium-rich impurity phases in the annealed solids (Y_8_V_2_O_17_ and Y_10_V_2_O_20_, Figure S13).

Hence, on the one hand, the YV2C0.5, YV2.5, and YV2.5C0.5 compositions are unsuitable for yielding chemically pure YVO_4_ solids for applications requiring high light outputs achieved by high temperature annealing. On the other hand, the intentional carbonation/hydroxylation procedures introduces structural and electronic effects in pristine particles leading to unconventional luminescent properties, such as Tb^3+^ emissions in YVO_4_ (Figure 5).

**Figure 5:**
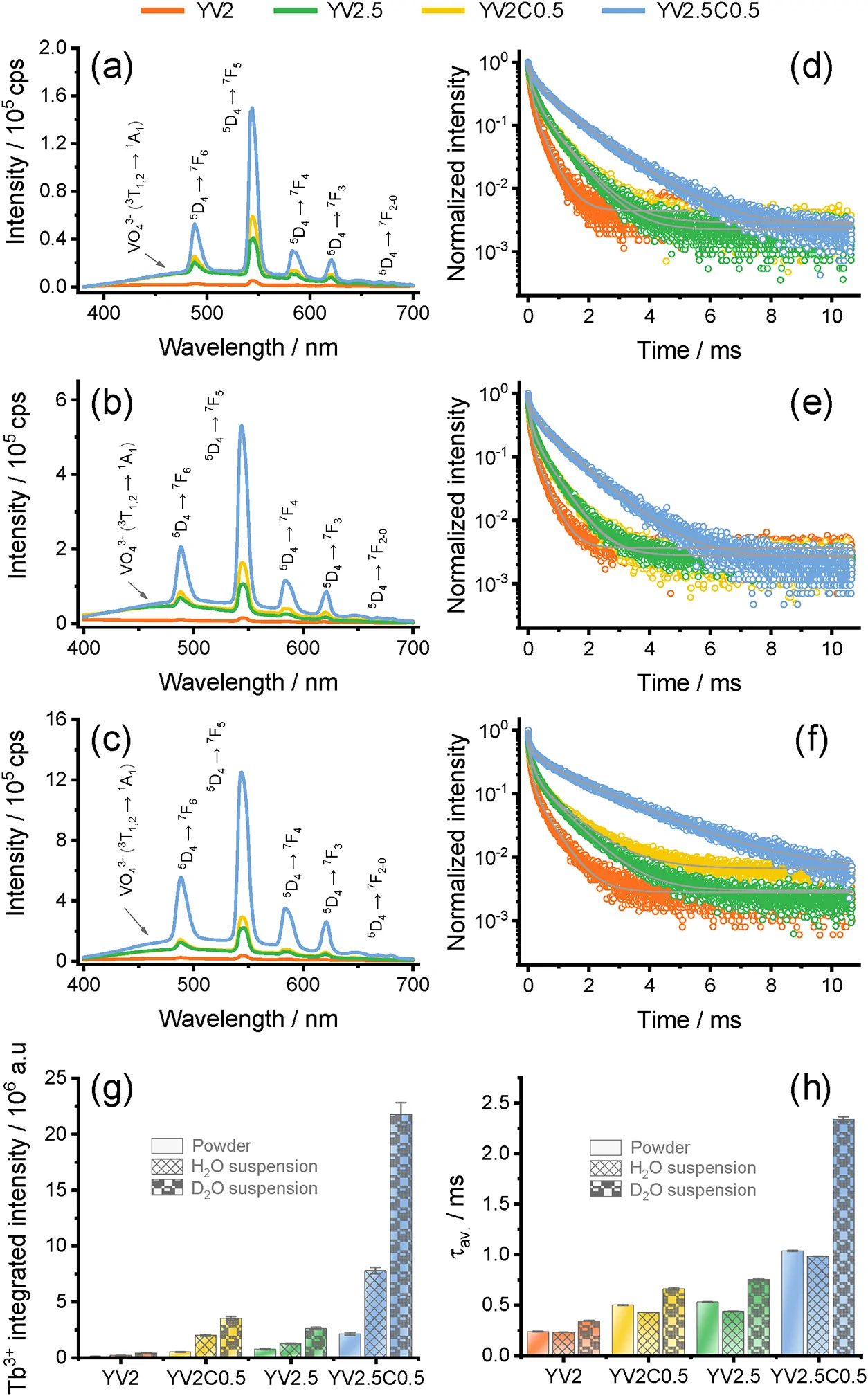
(a–c) Emission spectra (λ_exc_ = 320 nm) and (d–f) Tb^3+5^D_4_ emission decay curves (λ_exc_ = 320 nm, λ_em_ = 544 nm) of the Tb-doped YV2, YV2C0.5, YV2.5, and YV2.5C0.5 nanoparticles (Y_0.95_Tb_0.05_VO_4_) measured in (a,d) powder form and as 10 mg mL^–1^ dispersions in (b,e) H_2_O and (c, f) D_2_O. (g) Tb^3+^ integrated emission intensities and (h) average lifetimes (τ_av._) obtained from triexponential fitting of the experimental decay curves for the different samples. YV*n* and YV*n*C*x* indicate syntheses performed with *n* equivalents of NaOH and *x* equivalents of Na_2_CO_3_ with respect to the NaVO_3_ precursor.

As previously reported by us,^4^ the atypical luminescent behavior in YVO_4_:Tb^3+^ nanoparticles synthesized using Na_3_VO_4_ precursor stems from the shift of the metal–metal charge transfer (MMCT) Tb^4+^–V^4+^ state to higher energies compared to samples prepared by conventional solid-state synthesis. This shift is driven by the presence of oxygen defects and RE/V nonstoichiometry induced by CO_3_^2–^/OH^–^ species. We herein investigated the luminescence of intentionally hydroxylated/carbonated YVO_4_:Tb^3+^ particles (YV2C0.5, YV2.5, and YV2.5C0.5) in comparison to the chemically pure YV2 sample to demonstrate the tuneability of the light-output performance. We compared the emissions of the pure and modified nanoparticles at 25 °C as powders or colloids H_2_O and D_2_O. Excitation spectra (Figure S15) showed characteristic Tb^3+^ *f-f* absorptions (350 – 500 nm) and VO_4_^3–^ → Tb^3+^ energy transfer band due to the ^3^T_1,2_ ← ^1^A_1_ transitions of VO_4_^3–^ groups (230 – 350 nm). Under UV-excitation (λ_exc_ = 320 nm, Figure 5) or direct excitation at the ^5^D_4_ level (λ_exc_ = 486 nm, Figure S16) the particles showed the Tb^3+^ emissions comprising typical ^5^D_4_ → ^7^F_J_ (J = 6–0) bands with similar profiles for all particles. Under λ_exc_ = 320 nm, the Tb^3+^ are superimposed on a VO_4_^3–^ broadband emission (^3^T_1,2_ → ^1^A_1_), which extends from 380 to 700 nm (Figure S17). No signals from the ^5^D_3_ state were detected since this energy level lies above the MMCT quenching state and is prone to cross relaxation processes (*i.e*., ^5^D_3_ + ^7^F_6_ → ^5^D_4_ + ^7^F_0_) given the slightly high Tb^3+^ concentration (*i.e*. 5%). In addition, despite the possibility of strong surface quenching induced by H_2_O/OH groups in the ^5^D_4_ emitting level, the samples maintained their emissions with high signal-to-noise ratios even in water dispersions (Figure 5b).

The influence of hydroxylation and carbonation becomes evident when comparing the absolute emission intensities of the samples (Figure 5g and Figure S18, which shows spectra acquired in an integrating sphere). First, the chemically pure YV2:Tb^3+^ are expected to not show Tb^3+^ luminescence, as occurs for bulk YVO_4_:Tb^3+^ solids.^43–47^ However, the characteristics of this sample (*i.e*., enhanced defect concentration and structural distortions with respect to bulk) enable detection of Tb^3+^ emissions at very low intensities. Further hydroxylation of these particles as for the YV2.5:Tb^3+^ sample should result in even lower emission intensities due to enhanced vibronic quenching caused by the higher concentration of OH oscillators.^48^ Conversely, the hydroxylated particles exhibited improved emission intensities (*ca*. 3.5x higher considering integrated intensities) with respect to the chemically pure YV2:Tb^3+^ solids (Figure S18), which can be explained as due to the defect-induced shift of the MMCT state to higher energies.^4^ This demonstrates that the luminescence enhancement caused by the structural and electronic effects introduced by the intentional hydroxide impurities overcome the vibronic quenching processes. Carbonation of YV2:Tb^3+^ nanoparticles (*i.e*., YV2C0.5:Tb^3+^) results in slightly higher luminescence enhancement (*ca*. 6×, Figure S18) because of the same effects. In turn, the YV2.5C0.5:Tb^3+^ sample exhibits the highest emission intensity, showing that the simultaneous inclusion of hydroxide and carbonate species increases the energy barrier for the nonradiative MMCT quenching process,^4,46^ thus culminating in an emission enhancement of about 13 times with respect to the chemically pure YV2:Tb^3+^ sample (Figures 5g and S18). The shift of the MMCT states caused by compositional modification is further confirmed by the UV–vis absorption spectra of the solids (measured as diffuse reflectance, Figure S19), which show that the addition of CO_3_^2–^ and/or OH^–^ cause a hypsochromic shift of the barycenter of the absorption bands in UV. The low energy components (350–500 nm) of these UV bands are due to the MMCT transitions,^4^ so the decreased absorption intensities in this range after compositional alteration are a consequence of the shift of the MMCT to higher energies. These findings highlight the crucial role of structural and electronic modifications in tailoring the luminescent properties of YVO_4_:Tb^3+^ particles.

We examined the ^5^D_4_ luminescence lifetimes of the different Tb^3+^-doped particles as powders and as aqueous suspensions in H_2_O or D_2_O (Figure 5d–f). Although a single cation site is available for Tb^3+^ in the tetragonal YVO_4_ structure, the experimental decays fitted well triexponential functions, indicating that activator ions occupy multiple environments as commonly observed for nanosized vanadates and other host lattices.^49–51^ In the YV2, YV2C0.5, YV2.5, and YV2.5C0.5 samples, the Tb^3+^ ions are distributed between both the surface and the inner volume of the particles, with sites potentially displaying additional distortions caused by the different types of defects in these solids. The average ^5^D_4_ lifetimes (Figure 5h) were calculated considering an amplitude-weighted mean of the three lifetime components (Supporting Information), showing similar behaviors with respect to compositional changes and surface state of the particles (*i.e*., powder, H_2_O and D_2_O). From the compositional point of view, the results demonstrate that the intentional addition of carbonate and hydroxide impurities provide an increase of the ^5^D_4_ lifetimes in comparison to the YV2:Tb^3+^ sample. Even though a high concentration of defects may lead to additional angular distortions in the coordination sites favoring higher radiative decay probabilities, the increase in lifetimes is mostly due to the shift of the MMCT state toward higher energies, resulting in lower nonradiative decay rates for the ^5^D_4_ emitting state. While isolated modifications with carbonates (YV2C0.5) and hydroxides (YV2.5) cause a similar increase (*ca*. 2 times, Figure 5h) in the mean ^5^D_4_ lifetime, combined hydroxylation/carbonation (YV2.5C0.5) leads to increases of 4–7 times with respect to the chemically pure YV2 sample. This highlights the major impact of the defect engineering in the YVO_4_ particles in adjusting intrinsic nonradiative mechanisms to enhance Tb^3+^ luminescence intensities.

The effects of extrinsic quenching mechanisms were also examined, involving multiphonon relaxation of the ^5^D_4_ state by water molecules at the solid–liquid interface. First, mean lifetimes are slightly decreased in H_2_O suspensions with respect to powder samples, which is an expected consequence of increased multiphonon relaxation of the ^5^D_4_ state in water. Considering that the as-synthesized YV2, YV2C0.5, YV2.5, and YV2.5C0.5 powder particles show high amounts of adsorbed water, lifetimes in water were very similar and reduced to 85–95% of the values observed for powder particles (Figure 5h). Conversely, lifetimes in D_2_O provide an indication of the contribution of surface multiphonon quenching to the depopulation of the ^5^D_4_ state caused by exchangeable OH oscillators near to Tb^3+^ ion, which comprises surface hydroxide groups, coordinated water molecules, and closely diffusing water molecules.^48,52,53^ The results confirm that the surface quenching is not the dominant nonradiative mechanism for the YV2, YV2C0.5, YV2.5 samples, which show rather close ^5^D_4_ lifetimes for H_2_O or D_2_O suspensions (*i.e*., ∼1.5× higher in D_2_O, Figure 5h). In other words, nonradiative depopulation of the ^5^D_4_ state is dominated by the MMCT process in these cases. However, in the YV2.5C0.5 sample, the even higher energy shift of the MMCT states reduce the contribution of this nonradiative pathway, so the effects of OH/OD exchange become more evident. An increase of ∼2.4× in lifetimes from H_2_O to D_2_O suspensions is observed in this case. Hence, the results demonstrate that controlled hydroxylation and carbonation of yttrium vanadate particles is an effective strategy for fine adjusting the luminescent intensity of Tb^3+^ in this host lattice. This tuneability underscores the potential of these nanophosphors for advanced photonic applications, particularly in optical and (photo)­redox-based chemical and thermal luminescent sensing.

## Conclusions

Carbonate and hydroxide defects in YVO_4_ can enhance specific luminescent properties, as in the case of Tb^3+^ emissions, but can lead to the formation of contaminant crystalline phases upon annealing, as for the generation of Y_8_V_2_O_17_ at temperatures around 750 °C. The choice between high defect density for pristine particles and high structural purity after annealing is possible by the adequate selection of vanadate precursors combined with the intentional addition of CO_3_^2–^ and OH^–^. We hereby showed how morphological, structural and electronic effects introduced by these contaminants in YVO_4_ nanoparticles have a direct influence on their electronic properties. Unintentional carbonation of YVO_4_ nanoparticles occurs if highly basic Na_3_VO_4_ is used as the vanadate precursor, resulting in solids with high Y/V unbalance, which yields the impurity Y_8_V_2_O_17_ phase upon annealing. The thermal evolution provides an evaluation of the nanovoiding process caused by decomposition of CO_3_^2–^ and OH^–^ domains. An excellent correlation between observations involving *in situ* annealing in high-vacuum (TEM/SAED) and structural parameters obtained from *ex situ* thermal treatments in air (powder XRD) is observed. In addition, NaVO_3_ can be used as the vanadate precursor and be further combined to NaOH and Na_2_CO_3_ to yield chemically pure or controlled, intentionally modified YVO_4_ nanoparticles. The precise chemical control enabled the intentional hydroxylation and carbonation of YVO_4_:Tb^3+^ nanoparticles allows for tunable electronic structures that optimize their luminescent properties. The presence of OH^–^/CO_3_^2–^ groups shifts the MMCT Tb^4+^–V^4+^ quenching state to higher energies, enhancing the Tb^3+^ emissions by decreasing intrinsic nonradiative decay routes for depopulation of the ^5^D_4_ emitting state. The MMCT quenching is the dominant nonradiative pathway in chemically pure YVO_4_:Tb^3+^ nanoparticles, whereas carbonated/hydroxylated YVO_4_:Tb^3+^ nanoparticles show higher dependence on extrinsic surface quenching processes. Overall, the results highlight the major impacts of defect chemistry in YVO_4_ nanoparticles, providing a refined approach that holds great potential for producing high-quality REVO_4_ nanoparticles with tailored optical properties for improved efficiency in photonic applications.

## Supplementary Material



## Notes

X-ray diffractograms and results of data refinement, XPS spectra, FTIR spectra, *in situ* heating TEM images and SAED patterns, excitation and emission spectra, and analysis of Tb^3+5^D_4_ luminescence lifetimes (PDF)
