# Supplementary material for: Controlling Nanoscale Compositional Inhomogeneities to Enhance Tb3+ Luminescence in YVO4 Nanocrystals
Source: Inorg Chem. 2026 Aug 18;65(34):20029–39. doi: 10.1021/acs.inorgchem.6c03156 (PMC13536888; doi:10.1021/acs.inorgchem.6c03156)
Supplement: Supplementary file 1 [file ic6c03156_si_001.pdf]

SUPPORTING INFORMATION

Controlling Nanoscale Compositional Inhomogeneities to Enhance Tb^{3+} Luminescence in YVO_4 Nanocrystals

*Rafael V. Perrella,[a,b] Ileana Florea,[c,d] Eric Larquet,[b] Marc Walker,[f] Richard I. Walton,[g] Thierry Gacoin,[b] Paulo Cesar de Sousa Filho[a,]**

[a]Department of Inorganic Chemistry, Institute of Chemistry, Universidade Estadual de Campinas (Unicamp), R. Monteiro Lobato, 270, 13083-970, Campinas, São Paulo, Brazil
[b]Laboratoire de Physique de la Matière Condensée, Ecole Polytechnique, Institut Polytechnique de Paris, CNRS, Route de Saclay, 91128 Palaiseau, France
[c]Laboratory of Physics of Interfaces and Thin Films, Ecole Polytechnique, Institut Polytechnique de Paris, CNRS, 91228 Palaiseau, France
[d]Université Cote d'Azur (UniCA), CNRS, CRHEA, 06905 Sophia Antipolis Cedex, France
[e]Synchrotron SOLEIL, 91192, Gif-sur-Yvette, France.
[f]Department of Physics, University of Warwick, Coventry, CV4 7AL, United Kingdom
[g]Department of Chemistry, University of Warwick, Gibbet Hill Road, Coventry CV4 7AL, United Kingdom

*Corresponding author: pcsfilho@unicamp.br

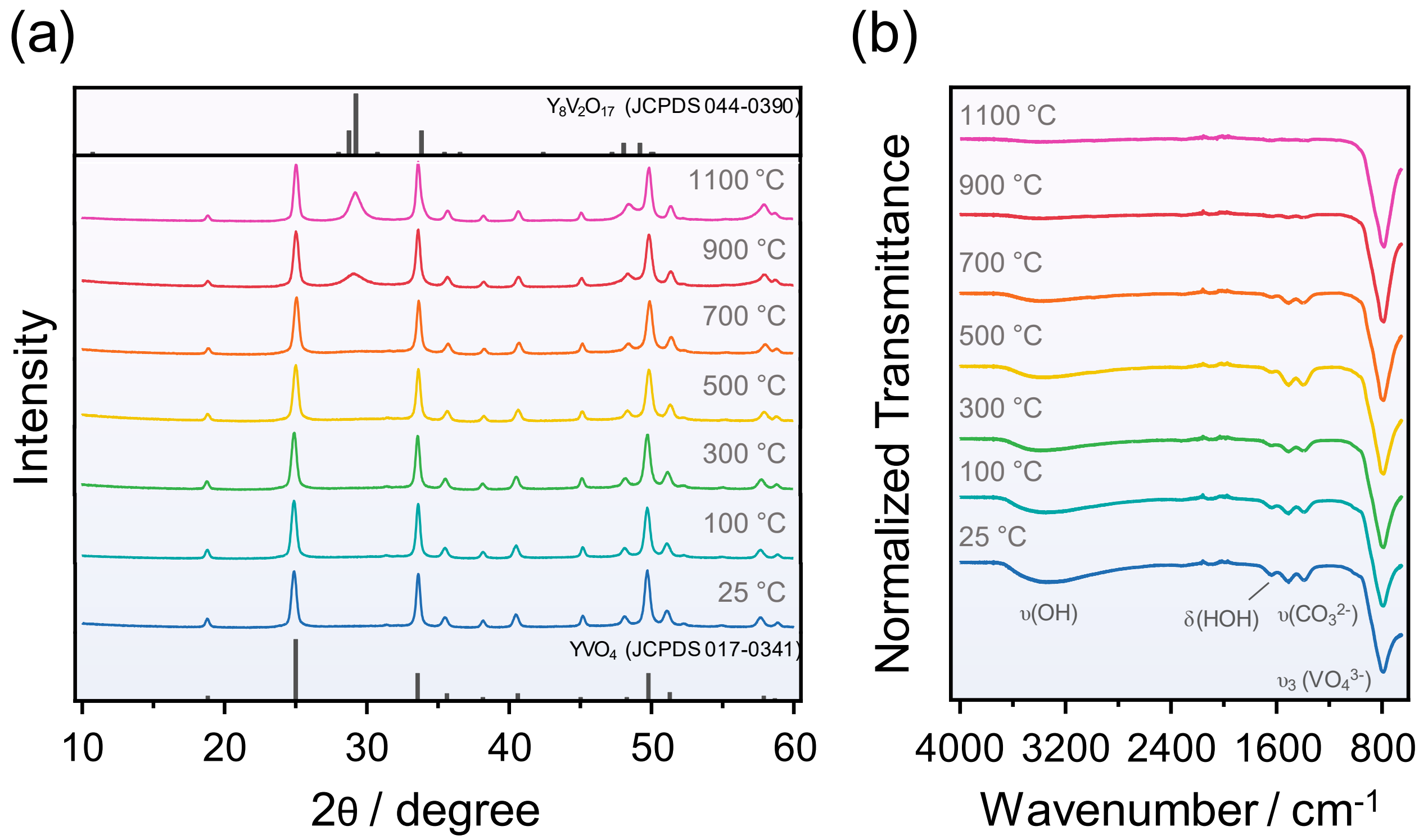

Figure S1. (a) Powder XRD patterns and (b) FTIR spectra of YVO_4 nanoparticles synthesized at 25 °C using Na_3VO_4 precursor and annealed in N_2 atmosphere between 100 to 1100 °C.

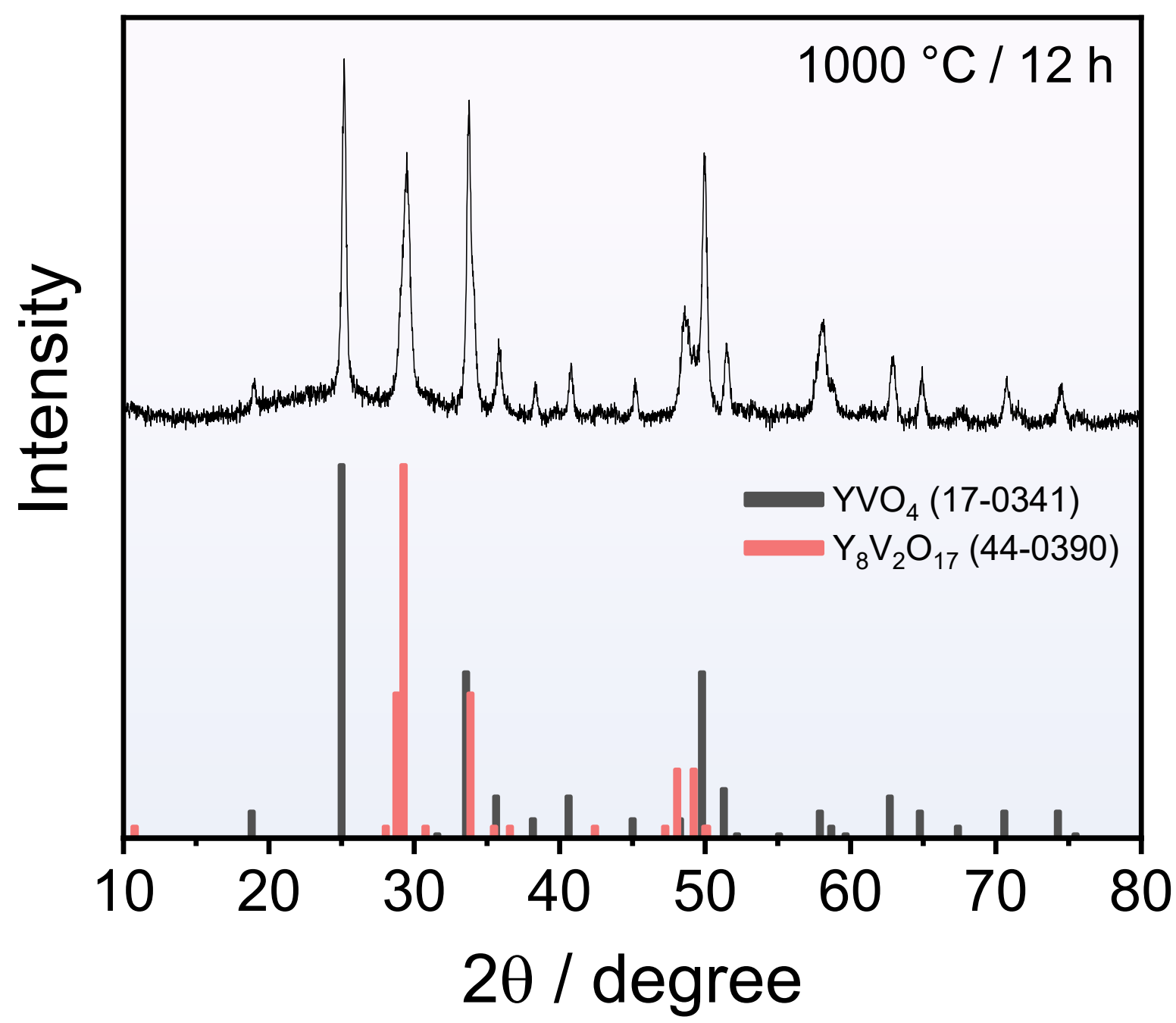

Figure S2. Powder XRD pattern of the YVO_4 particles prepared at 25 °C using Na_3VO_4 precursor and annealed at 1000 °C for 12 h. The experimental diffractogram is compared to the YVO_4 (JCPDS 17-0341, black) and $Y_8V_2O_{17}$ (JCPDS 44-0390, red) standards.

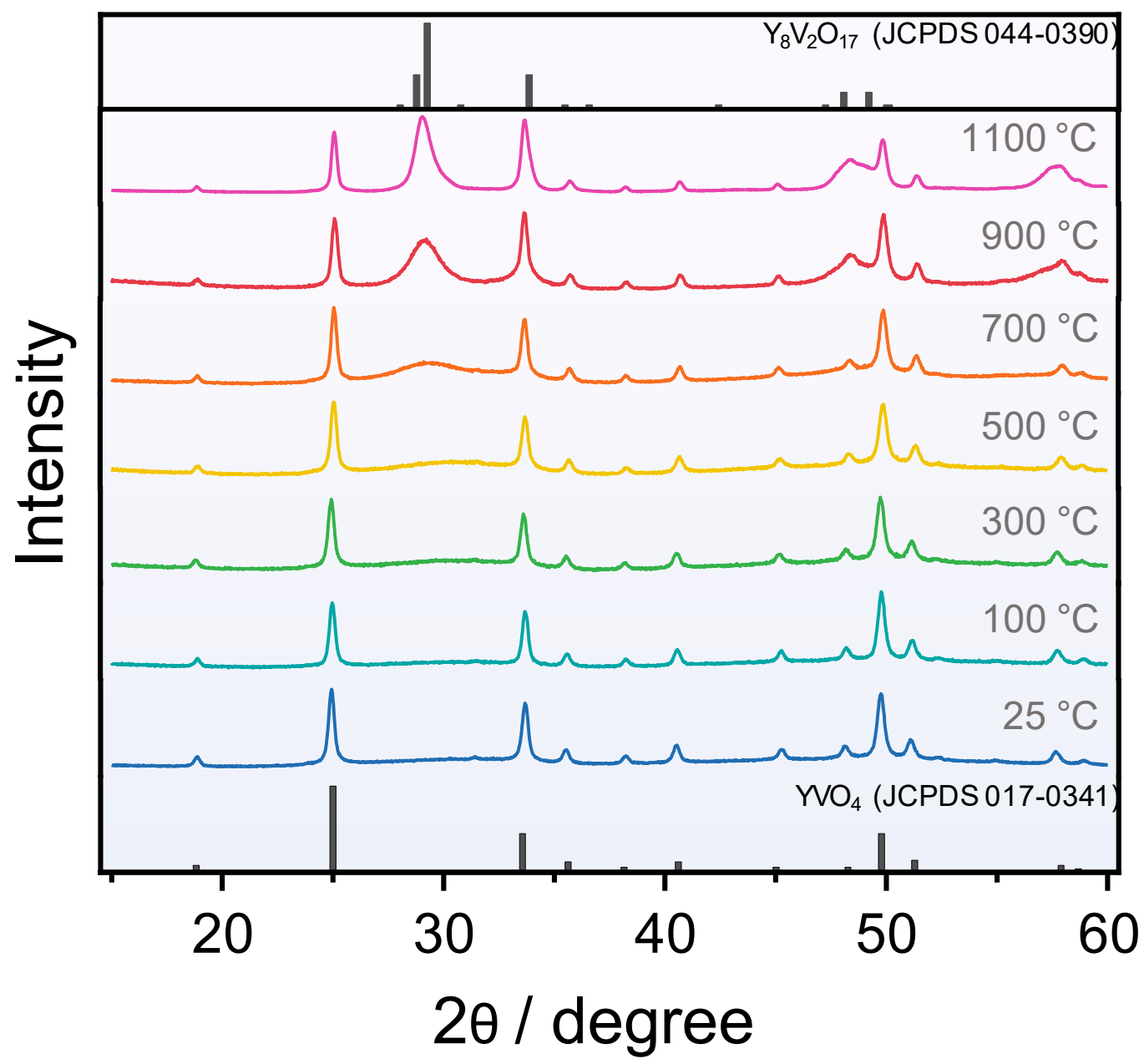

Figure S3. Powder XRD patterns of YVO_4 nanoparticles synthesized at 25 °C using a two-year-old opened Na_3VO_4 flask and annealed in N_2 atmosphere between 100 to 1100 °C.

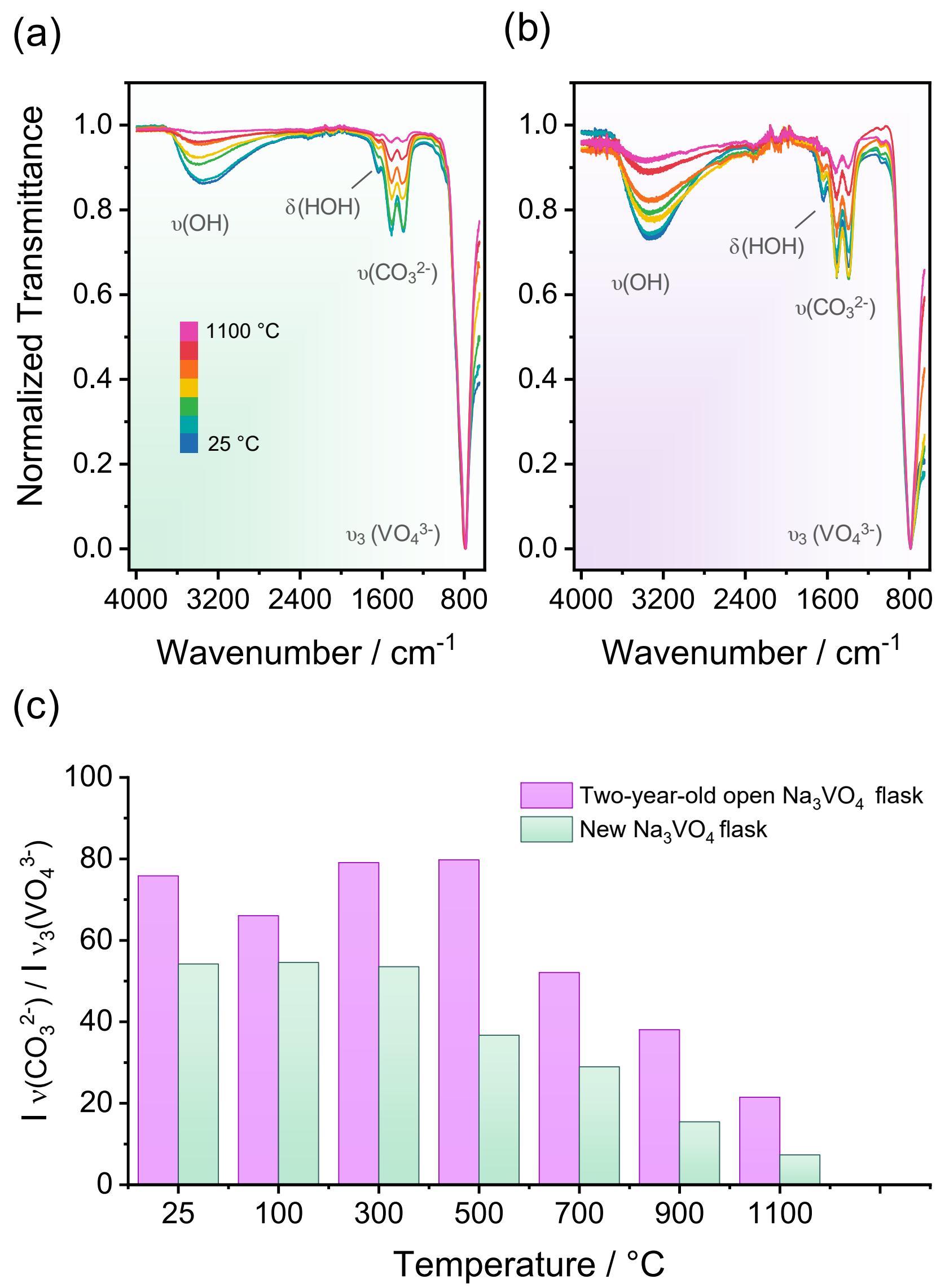

Figure S4. FTIR spectra of pristine (25 °C) and annealed YVO_4 nanoparticles synthesized using a (a) new and (b) two-year-old opened Na_3VO_4 flask. (c) Thermal evolution of the integrated intensity ratio involving the carbonate [$\nu(CO_3^{2-})$, 1510–1380 cm^{-1}] and vanadate [$\nu_3(VO_4^{3-})$, 950–650 cm^{-1}] stretching modes.

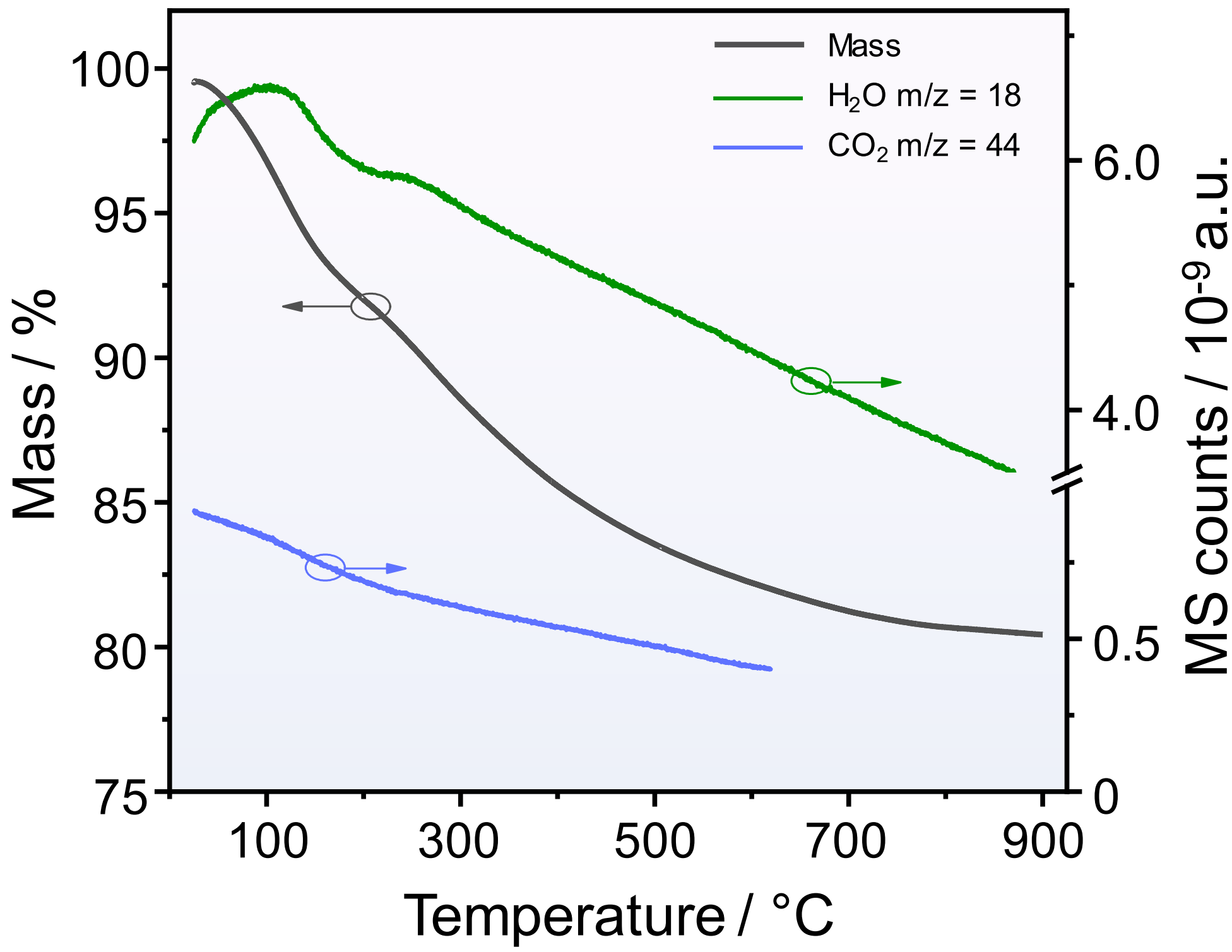

Figure S5. TGA (black line) and mass spectrometry analyses of gases evolved from YVO_4 particles synthesized at 25 °C using Na_3VO_4 precursor, highlighting the m/z = 18 (green, H_2O) and m/z = 44 (blue, CO_2) signals.

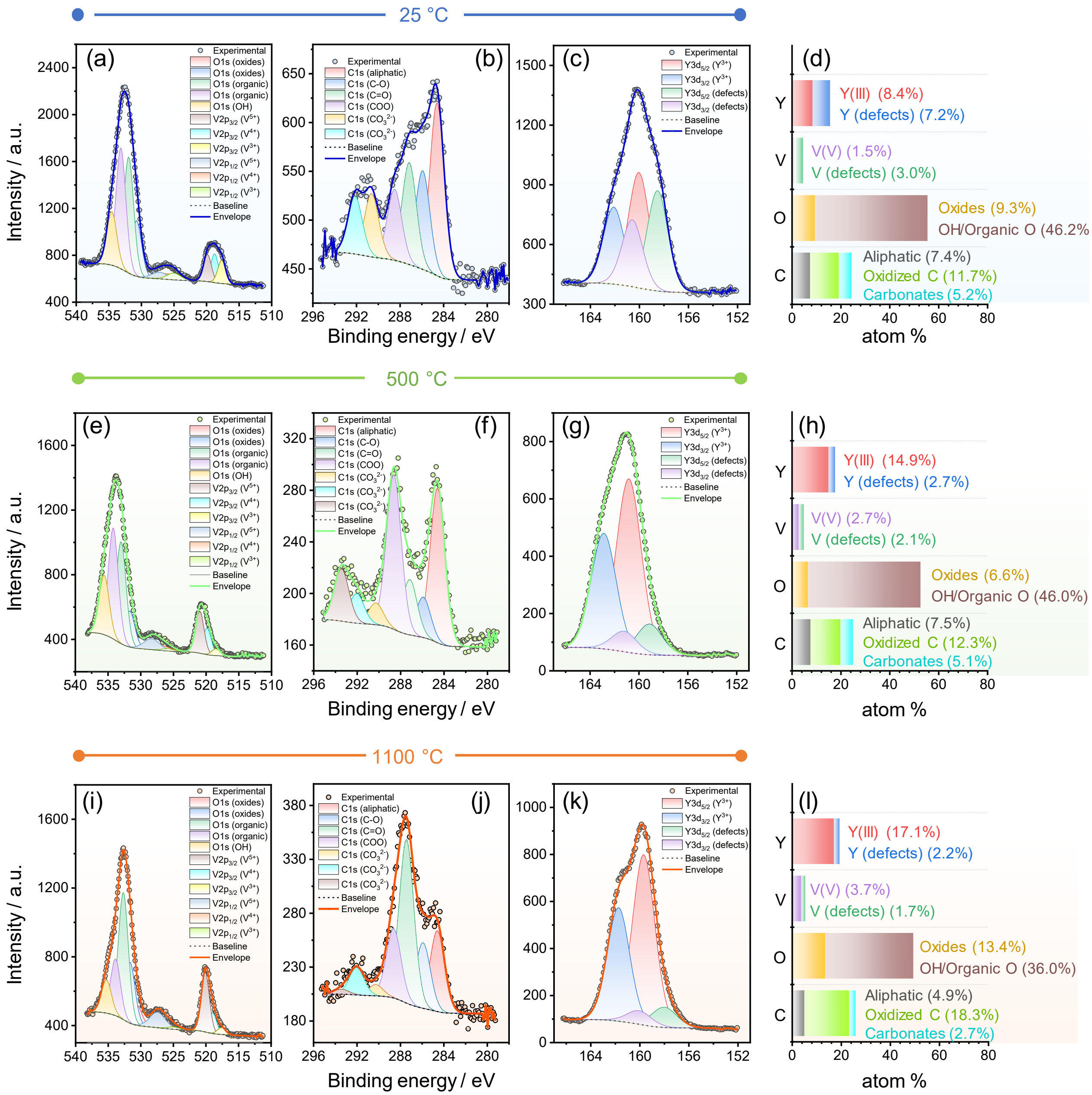

Figure S6. XPS spectra of YVO_4 nanoparticles synthesized at (a-c) 25 °C using Na_3VO_4 precursor and annealed at (e-g) 500 °C and (i-k) 1100 °C, showing the (a,e,i) O1s and V2p, (b,f,j) C1s, and (c,g,k) Y3d peaks. (d,h,l) Compositional results (atom %) obtained from XPS spectra.

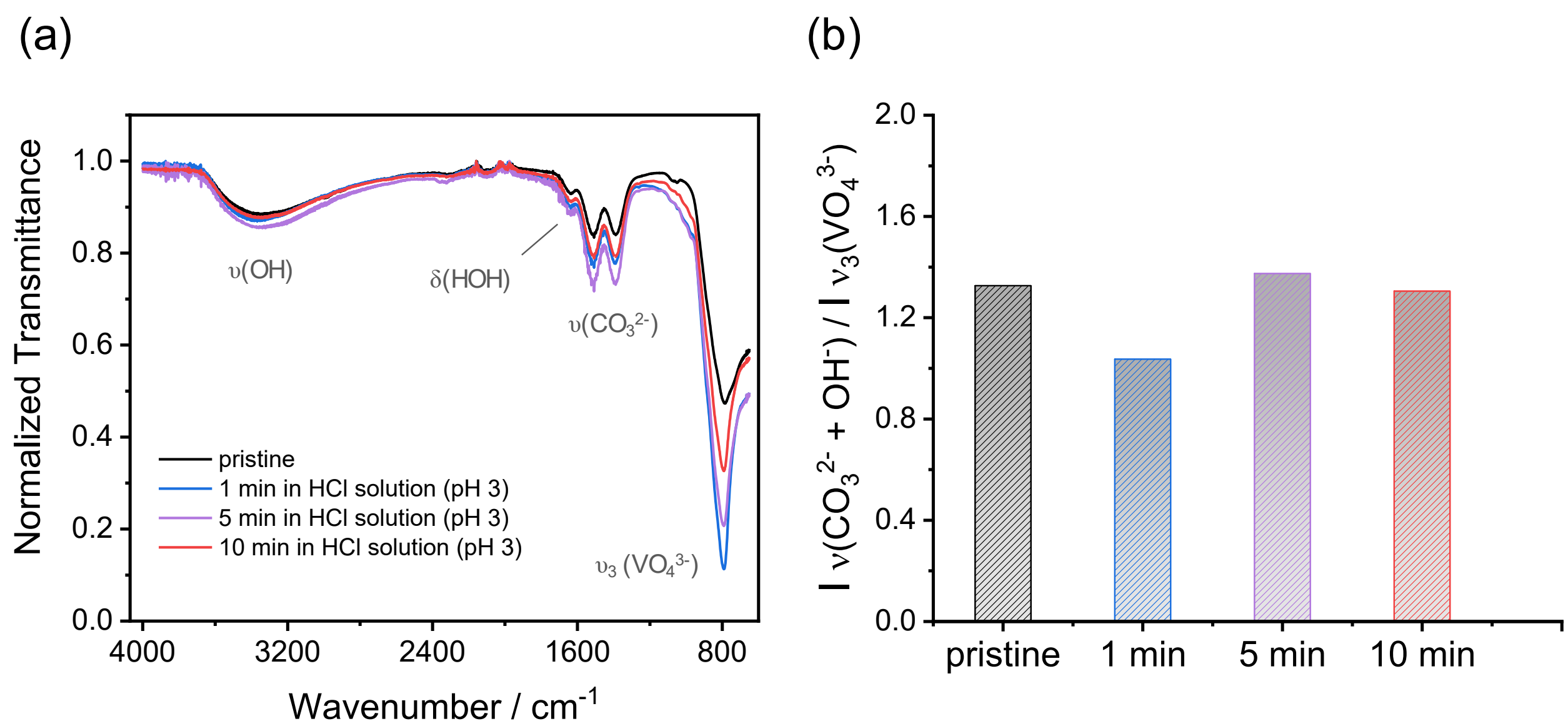

Figure S7. (a) FTIR spectra of YVO_4 nanoparticles synthesized using Na_3VO_4 precursor before (pristine, black line) and after acid treatment with an HCl solution (pH=3). All samples were dried at 60 °C overnight prior to spectrum acquisition. (b) Integrated intensity ratio of the carbonate/hydroxyl [$\nu(CO_3^{2-})/\nu(OH)$] and vanadate [$\nu_3(VO_4^{3-})$] stretching modes as a function of acid treatment time. The results indicate a minimal effect of the acid solution on the removal of hydroxyl/carbonate species from the solids.

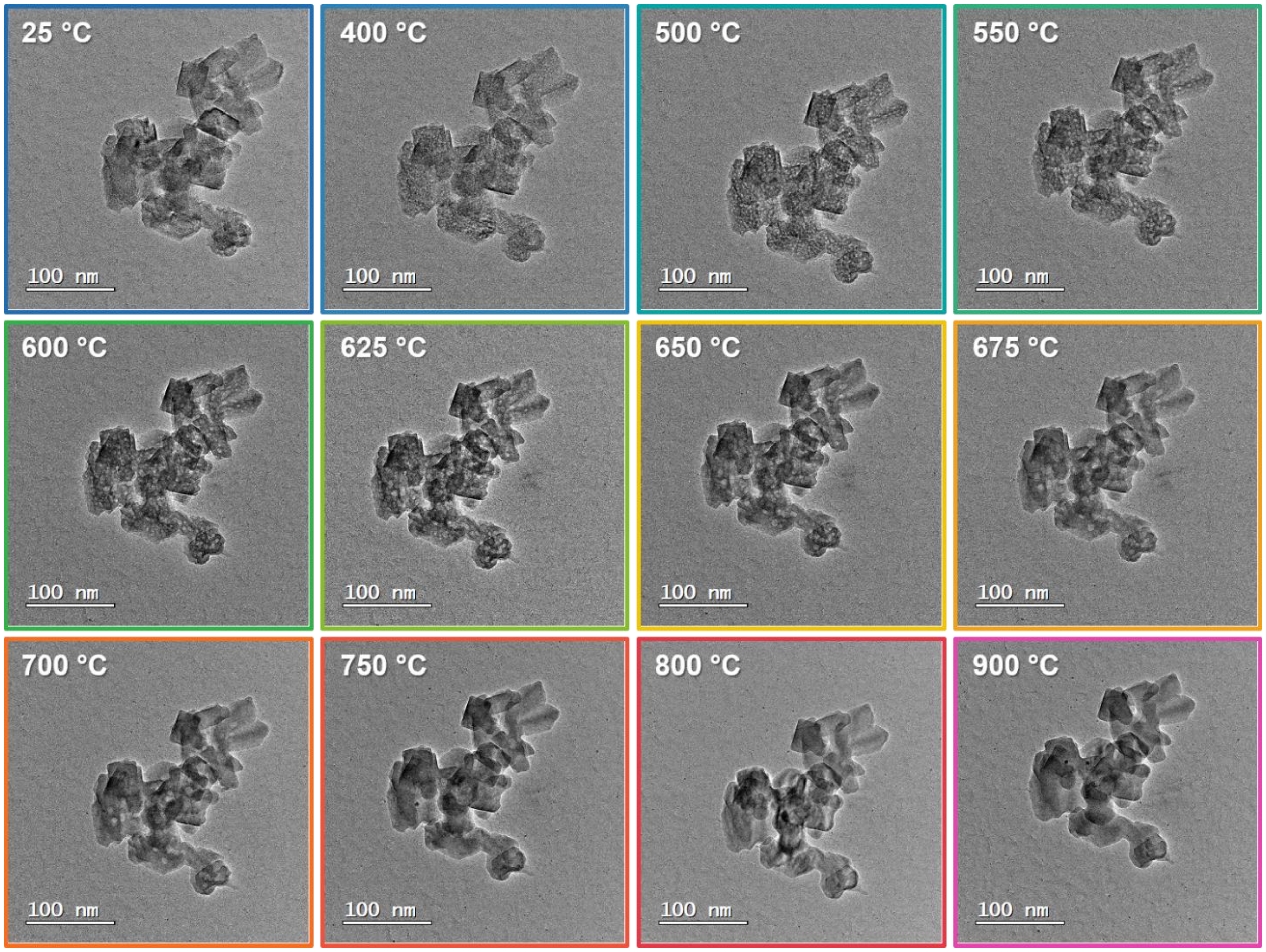

Figure S8. *In situ* heating TEM images of YVO_4 nanoparticles synthesized using Na_3VO_4 precursor. This sample region is designated as zone 1. Selected images are also shown in the Figure 2 of the main text.

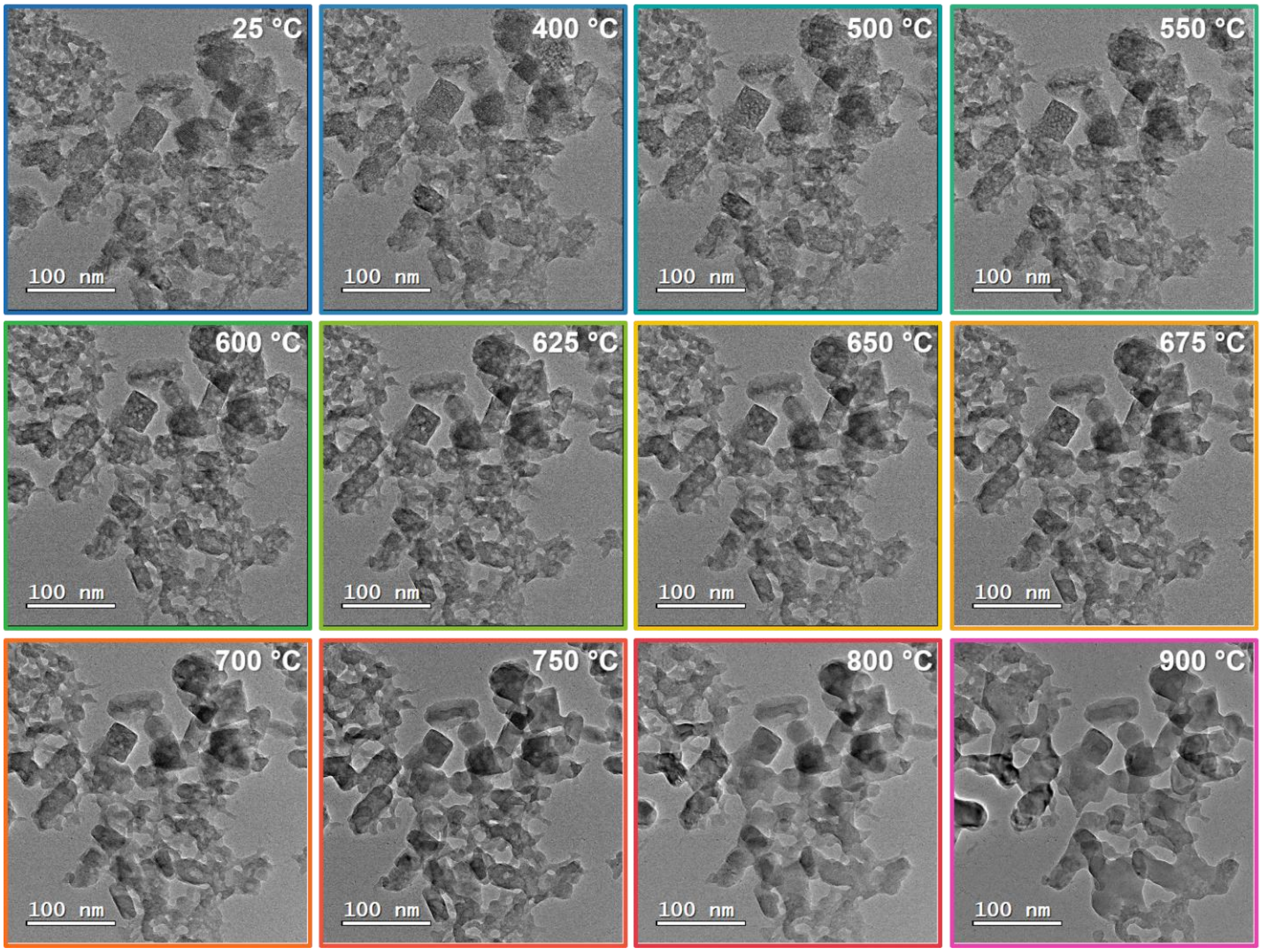

Figure S9. *In situ* heating TEM images of YVO_4 nanoparticles synthesized using Na_3VO_4 precursor. This sample region is designated as zone 2.

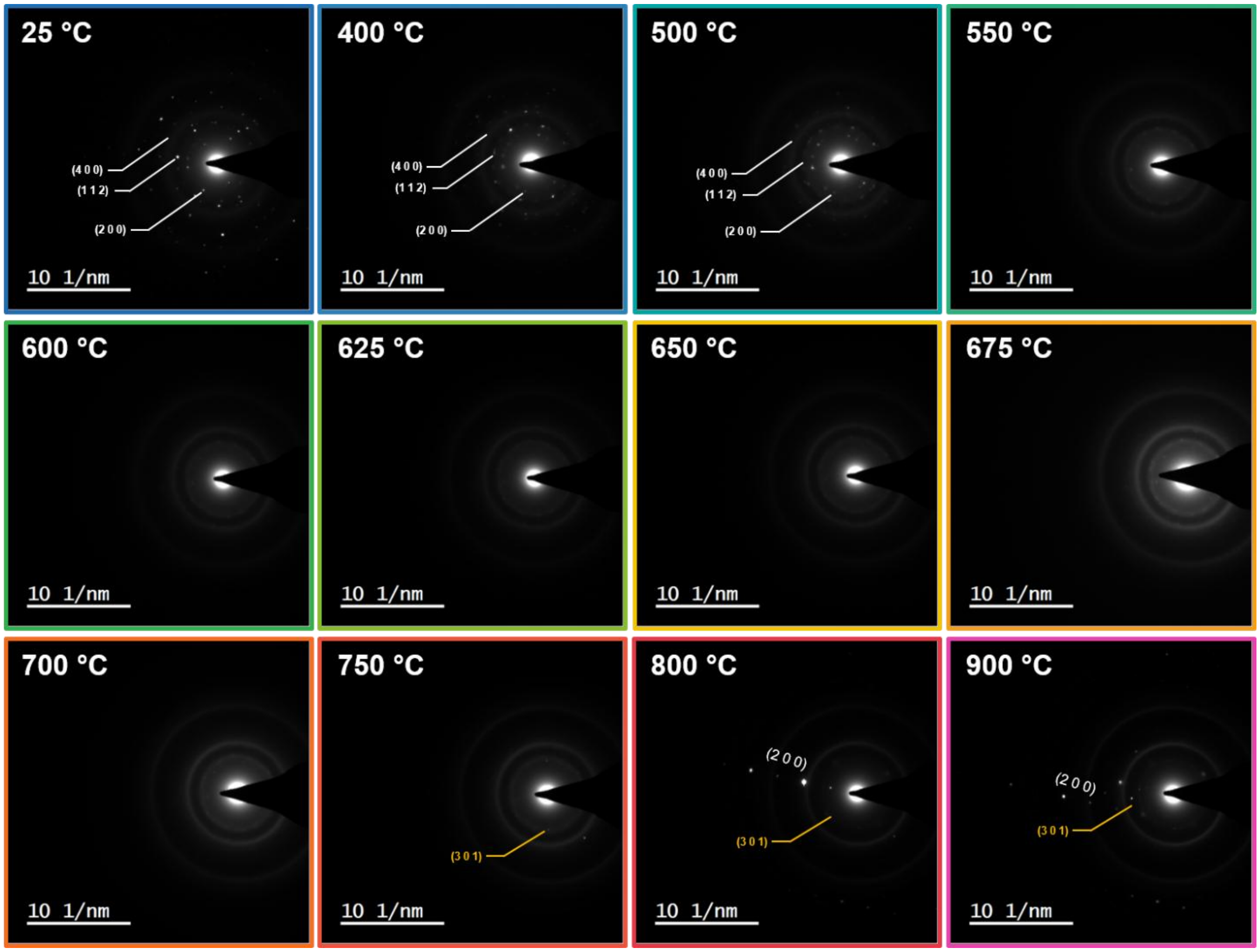

Figure S10. *In situ* heating SAED images of YVO_4 nanoparticles synthesized at 25 °C using Na_3VO_4 precursor. This sample region is designated as zone 1. The characteristic (4 0 0), (1 1 2), and (2 0 0) crystalline planes (white) of the tetragonal YVO_4 phase and the (3 0 1) plane (yellow) of the tetragonal $Y_8V_2O_{17}$ phase are indicated. Selected images are shown in the Figure 2 of the main text.

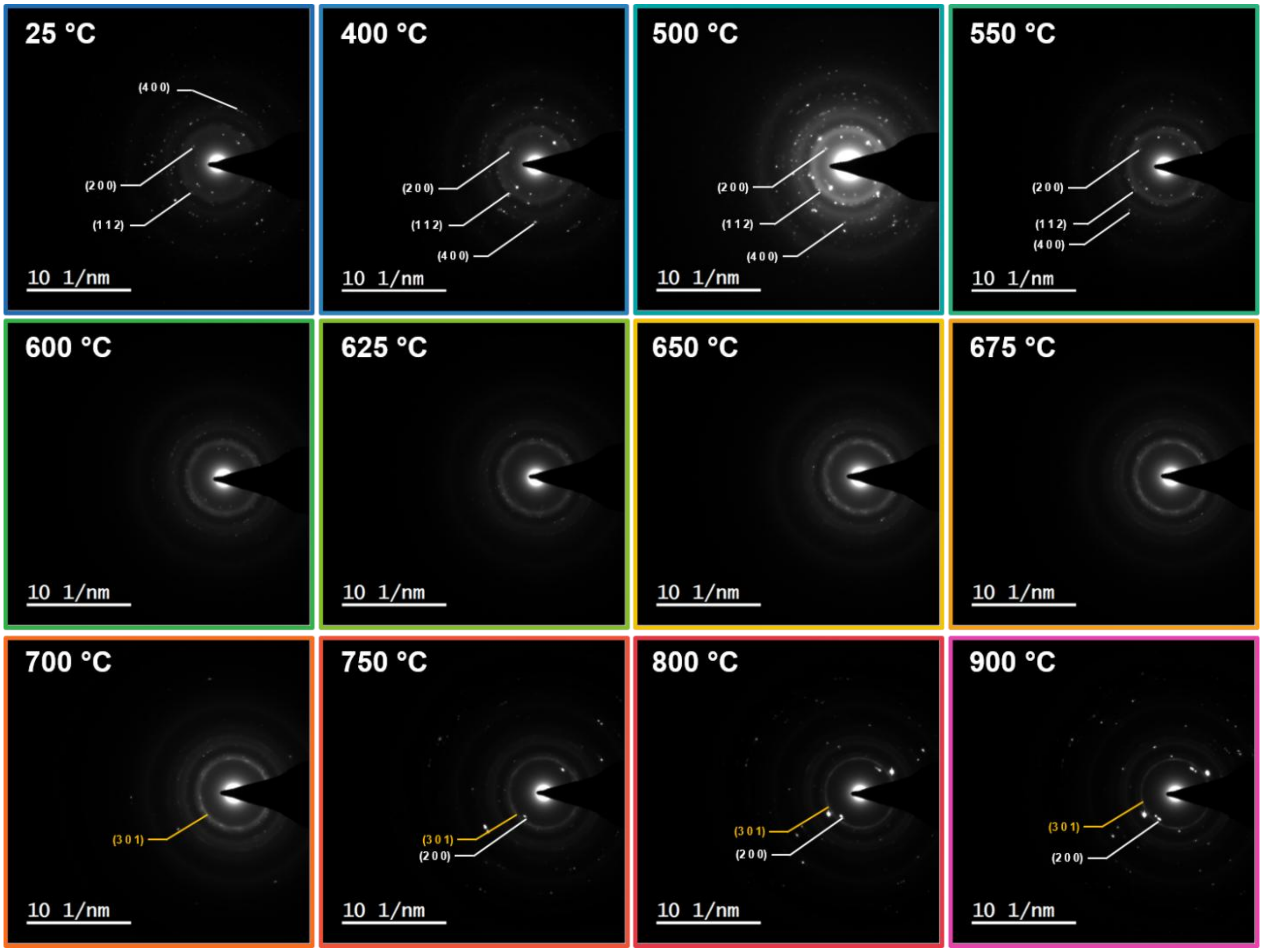

Figure S11. *In situ* heating SAED images of YVO_4 nanoparticles synthesized at 25 °C using Na_3VO_4 precursor. This sample region is designated as zone 2. The characteristic (4 0 0), (1 1 2), and (2 0 0) crystalline planes (white) of the tetragonal YVO_4 phase and the (3 0 1) plane (yellow) of the tetragonal $Y_8V_2O_{17}$ phase are indicated.

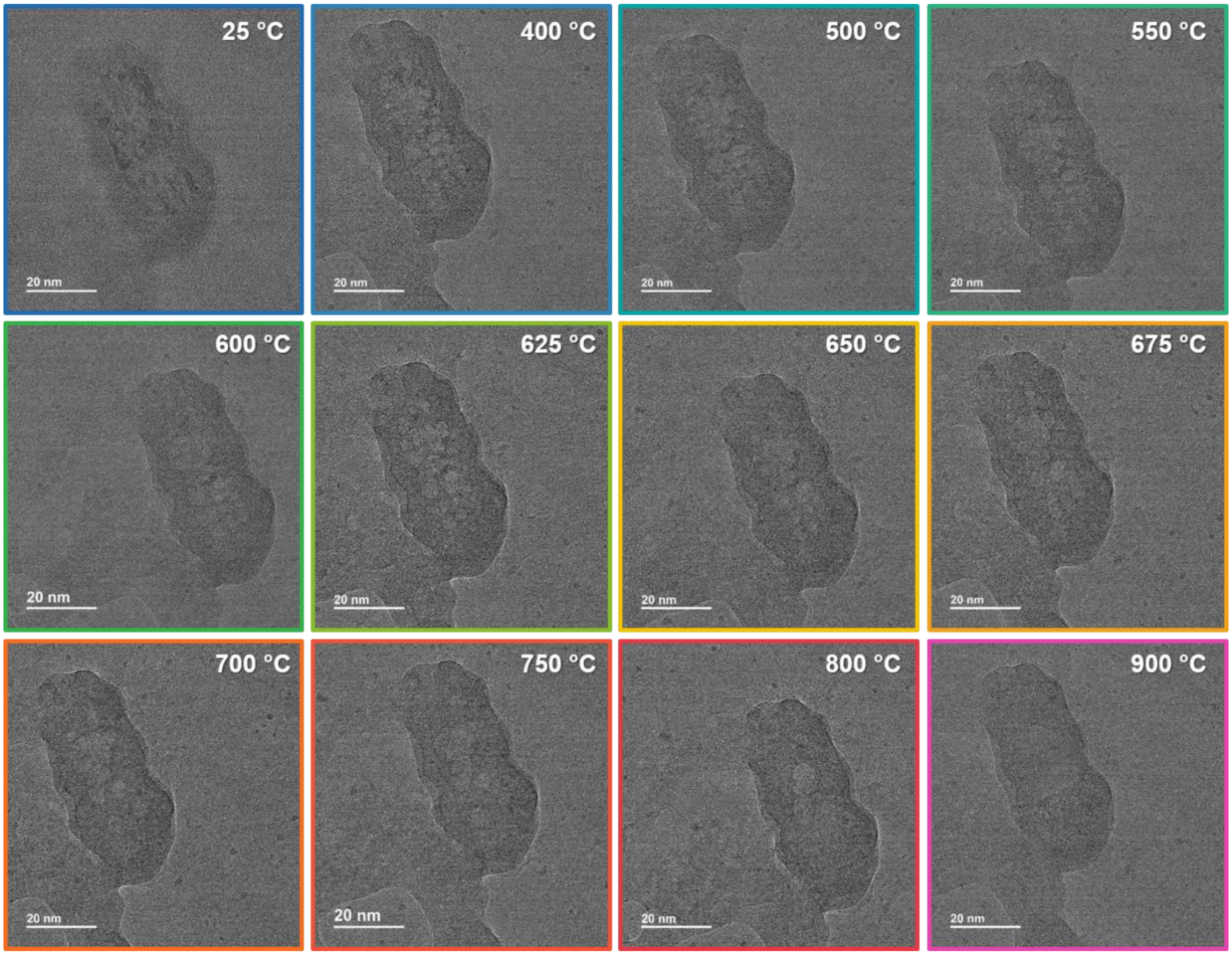

Figure S12. *In situ* heating high-resolution TEM images of YVO_4 nanoparticles synthesized at 25 °C using Na_3VO_4 precursor.

Table S1. Lattice parameters (*a* and *c*), unit cell volume (V), average coherence length (L_C), and microstrain (ε) values for the as-synthesized (25 °C) and annealed YVO_4 nanoparticles synthesized using Na_3VO_4 precursor. R_{wp}, R_p, and R_{exp} correspond to the quality fitting parameters obtained in the refinements. All diffraction peaks of the spurious phases were considered as background

Calcination temperature	*a* / Å	*c* / Å	V / $Å^3$	L_C / nm	ε x 10^{-3}	R_{wp}	R_p	R_{exp}
25 °C	7.152 ± 0.003	6.259 ± 0.002	320.1 ± 0.2	38.2 ± 0.4	0.68 ± 0.05	0.053	0.037	0.021
100 °C	7.150 ± 0.002	6.258 ± 0.001	319.9 ± 0.2	45.2 ± 0.7	1.38 ± 0.03	0.042	0.031	0.022
300 °C	7.132 ± 0.004	6.263 ± 0.004	318.6 ± 0.3	37.5 ± 2.3	1.45 ± 0.39	0.045	0.032	0.022
500 °C	7.107 ± 0.002	6.273 ± 0.002	316.8 ± 0.2	35.7 ± 0.8	1.14 ± 0.20	0.042	0.033	0.027
700 °C	7.106 ± 0.003	6.280 ± 0.003	317.1 ± 0.1	35.8 ± 1.3	1.61 ± 0.06	0.054	0.040	0.026
900 °C	7.114 ± 0.004	6.286 ± 0.003	318.1 ± 0.1	39.1 ± 2.5	1.33 ± 0.45	0.051	0.039	0.032
1100 °C	7.117 ± 0.002	6.292 ± 0.002	318.7 ± 0.1	76.3 ± 6.2	1.07 ± 0.09	0.067	0.048	0.025

Table S2. Lattice parameters (*a* and *c*), unit cell volume (V), average coherence length (L_C), and microstrain (ε) values for the as-synthesized and annealed YVO_4 nanoparticles synthesized using $NaVO_3$ precursor supplemented with known amounts of NaOH and Na_2CO_3 (YV*n* and YV*n*C*x* compositions). R_{wp}, R_p, and R_{exp} correspond to the quality fitting parameters obtained in the refinements. All refinements considered only the diffraction signals of the YVO_4 phase. Coherence lengths for the annealed solids exceeded 1000 nm and were therefore excluded from the analysis

Sample	*a* / Å	*c* / Å	V / Å^3	L_C / nm	ε x 10^{-3}	R_{wp}	R_p	R_{exp}
As-synthesized (25 °C)								
YV2	7.140 ± 0.002	6.306 ± 0.002	321.4 ± 0.2	7.1 ± 0.4	0.18 ± 0.03	0.080	0.062	0.051
YV2C0.5	7.139 ± 0.001	6.280 ± 0.002	320.0 ± 0.1	7.0 ± 0.3	5.39 ± 0.02	0.062	0.046	0.048
YV2.5	7.137 ± 0.001	6.272 ± 0.001	319.5 ± 0.1	23.6 ± 1.9	0.11 ± 0.01	0.113	0.086	0.050
YV2.5C0.5	7.164 ± 0.002	6.274 ± 0.002	322.0 ± 0.2	5.3 ± 0.5	5.03 ± 0.34	0.061	0.047	0.044
Annealed at 1000 °C								
YV2	7.118 ± 0.001	6.299 ± 0.001	319.2 ± 0.1	-	-	0.160	0.127	0.064
YV2C0.5	7.121 ± 0.001	6.295 ± 0.001	319.3 ± 0.1	-	-	0.365	0.268	0.051
YV2.5	7.125 ± 0.001	6.298 ± 0.002	319.8 ± 0.1	-	-	0.321	0.226	0.057
YV2.5C0.5	7.127 ± 0.001	6.296 ± 0.001	319.8 ± 0.1	-	-	0.449	0.336	0.059

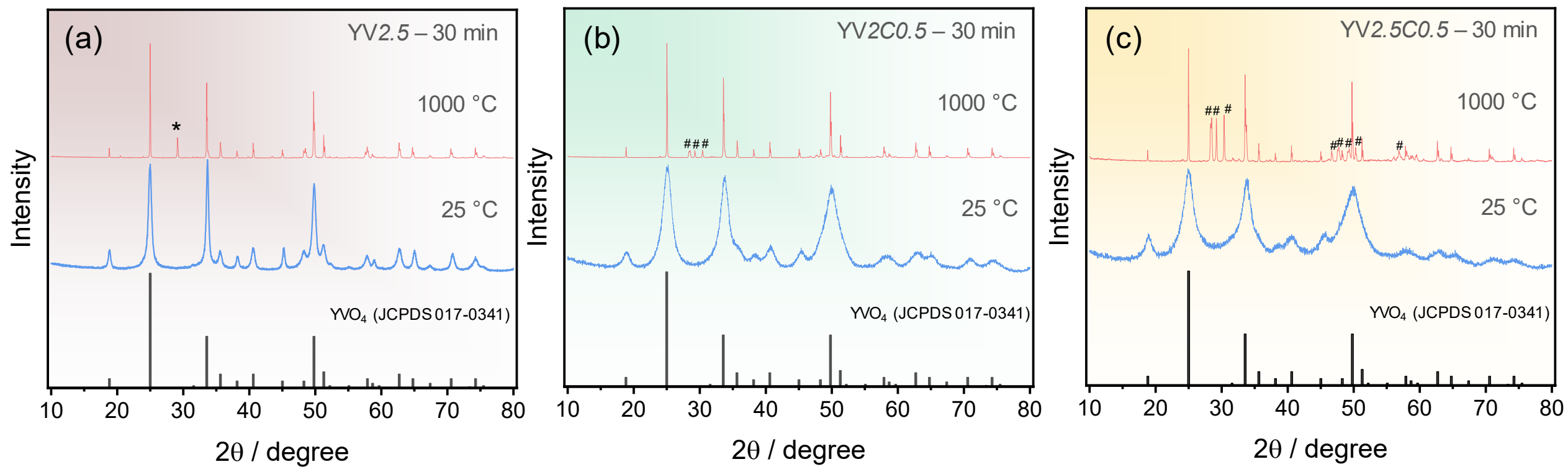

Figure S13. Powder XRD patterns of (a) YV2.5, (b) YV2C0.5, and (c) YV2.5C0.5 samples synthesized at 25 °C using $NaVO_3$ precursor and annealed at 1000 °C. YV*n* and YV*n*C*x* notations refer to the preparation of YVO_4 nanoparticles using *n* equivalents of NaOH and *x* equivalents of Na_2CO_3 relative to the metavanadate precursor. The * and # symbols denote the diffraction peaks assigned to the $Y_8V_2O_{17}$ (JCPDS 00-0000) and $Y_{10}V_2O_{20}$ (JCPDS 00-0000) secondary phases, respectively. XRD patterns of the as-prepared and annealed YV2 sample (chemically pure YVO_4) are shown in Figure 1c of the main text.

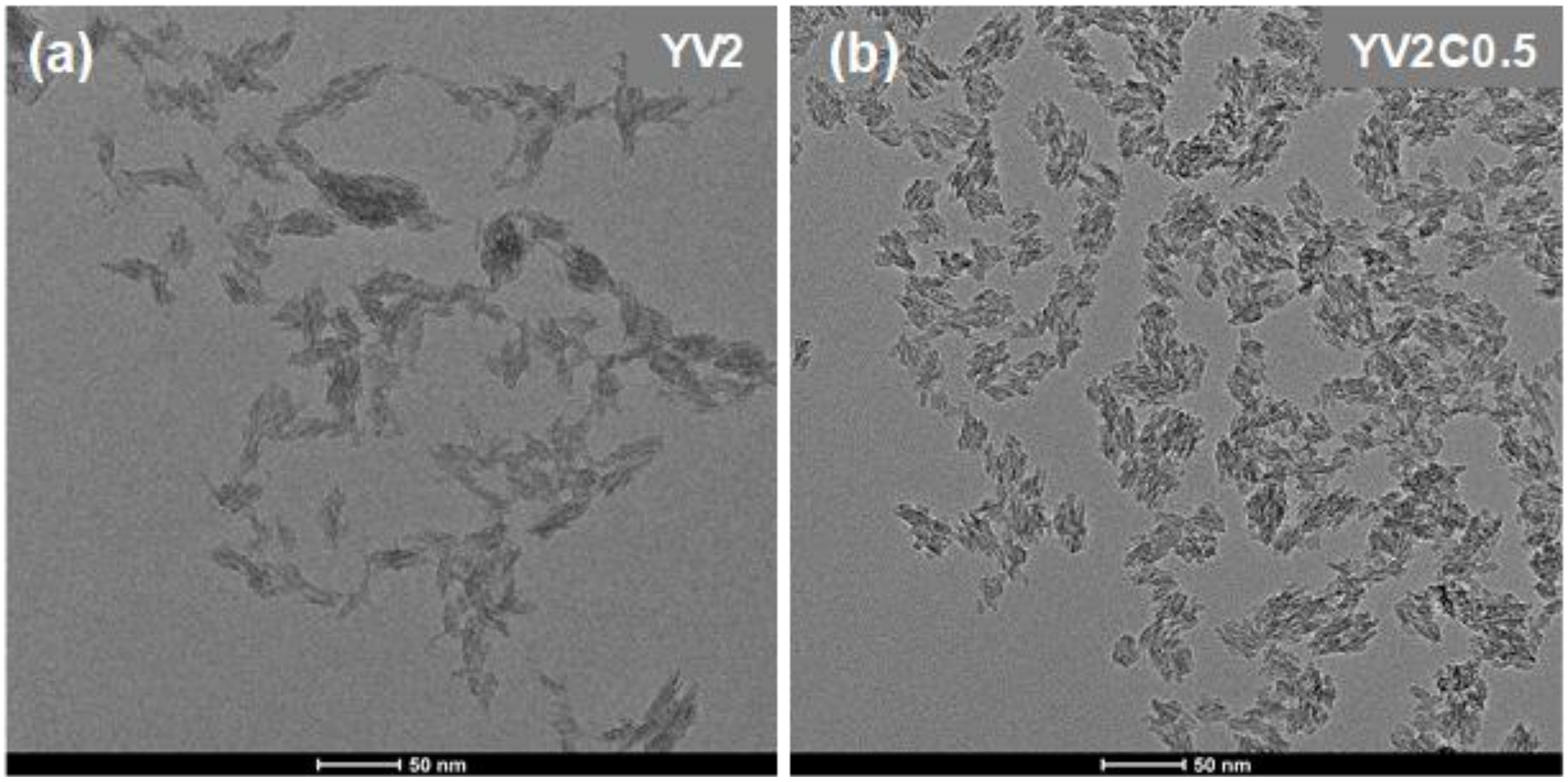

Figure S14. TEM images of (a) YV2 and (b) YV2C0.5 particles prepared at 25 °C using $NaVO_3$ precursor. YV*n* and YV*n*C*x* labels indicate syntheses performed with *n* equivalents of NaOH and *x* equivalents of Na_2CO_3 with respect to the metavanadate precursor.

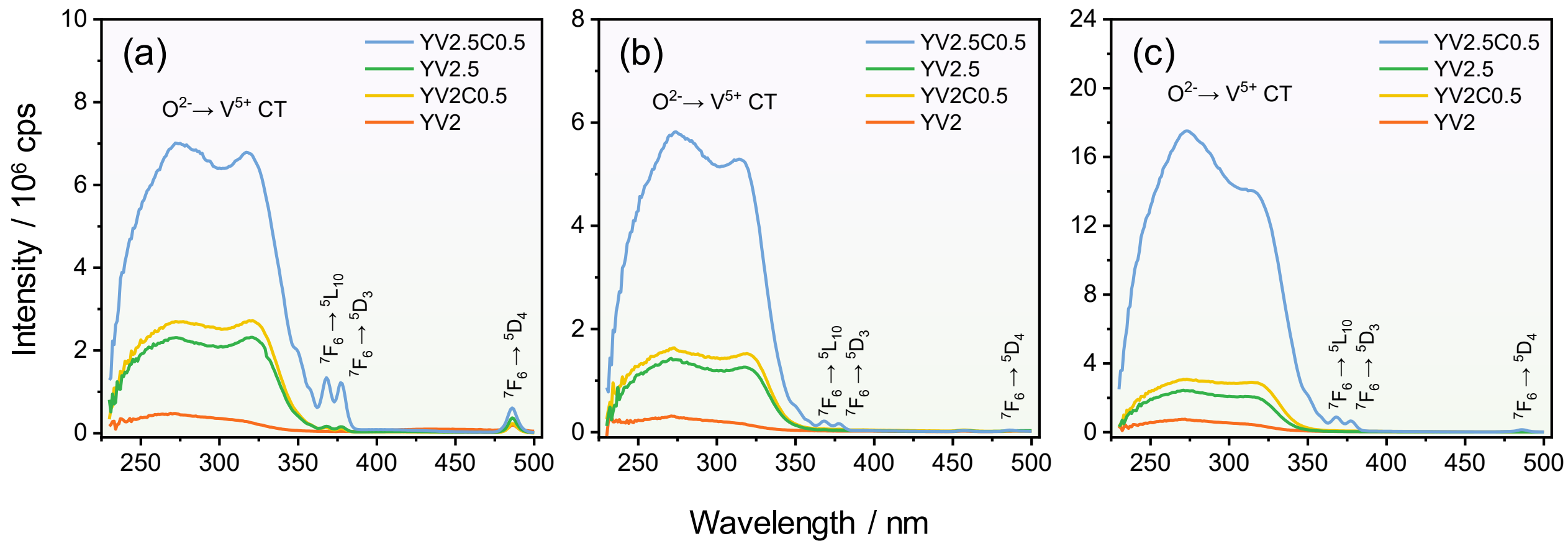

Figure S15. Excitation spectra (λ_{em} = 544 nm) of Tb-doped YV2, YV2C0.5, YV2.5, and YV2.5C0.5 nanoparticles ($(Y_{0.95}Tb_{0.05})VO_4$) measured in (a) powder form and as 10 mg mL^{-1} dispersions in (b) H_2O and (c) D_2O. YV*n* and YV*n*C*x* labels indicate syntheses performed with *n* equivalents of NaOH and *x* equivalents of Na_2CO_3 with respect to the $NaVO_3$ precursor.

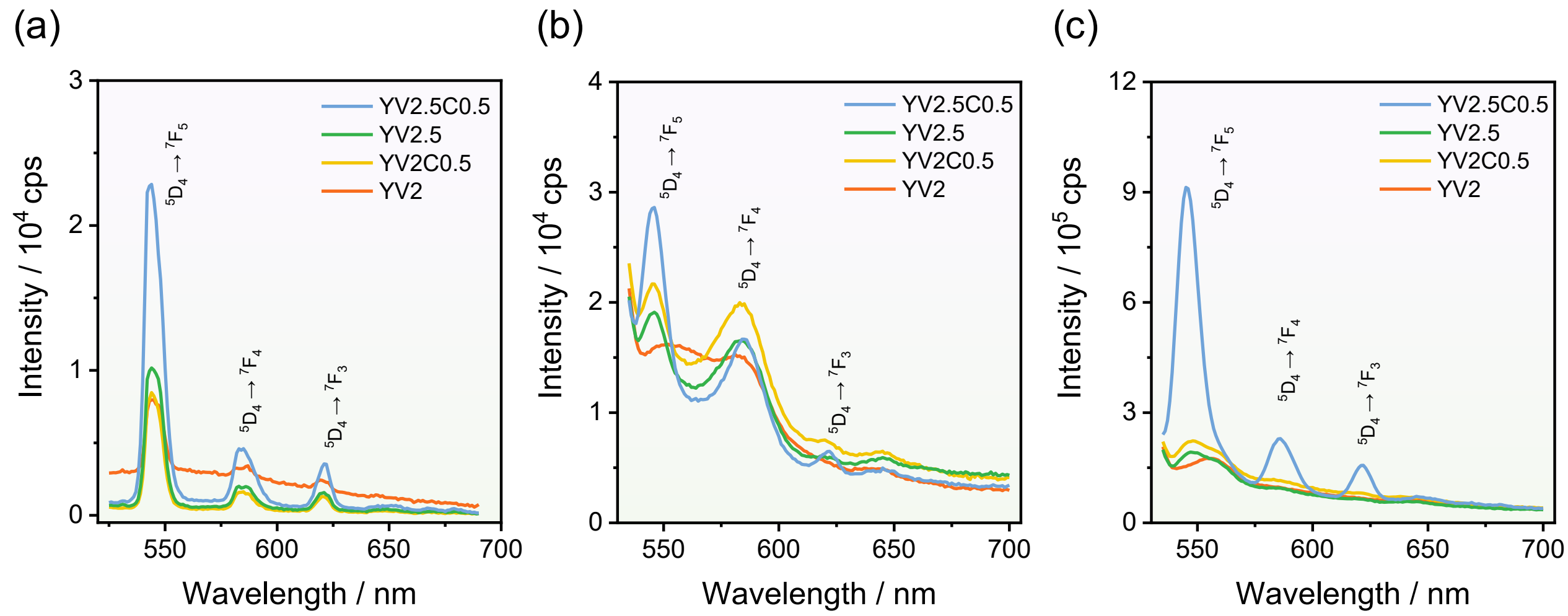

Figure S16. Emission spectra (λ_{exc} = 486 nm) of Tb-doped YV2, YV2C0.5, YV2.5, and YV2.5C0.5 nanoparticles $(Y_{0.95}Tb_{0.05})VO_4$ measured in (a) powder form and as 10 mg mL^{-1} dispersions in (b) H_2O and (c) D_2O). YV*n* and YV*n*C*x* labels indicate syntheses performed with *n* equivalents of NaOH and *x* equivalents of Na_2CO_3 with respect to the $NaVO_3$ precursor.

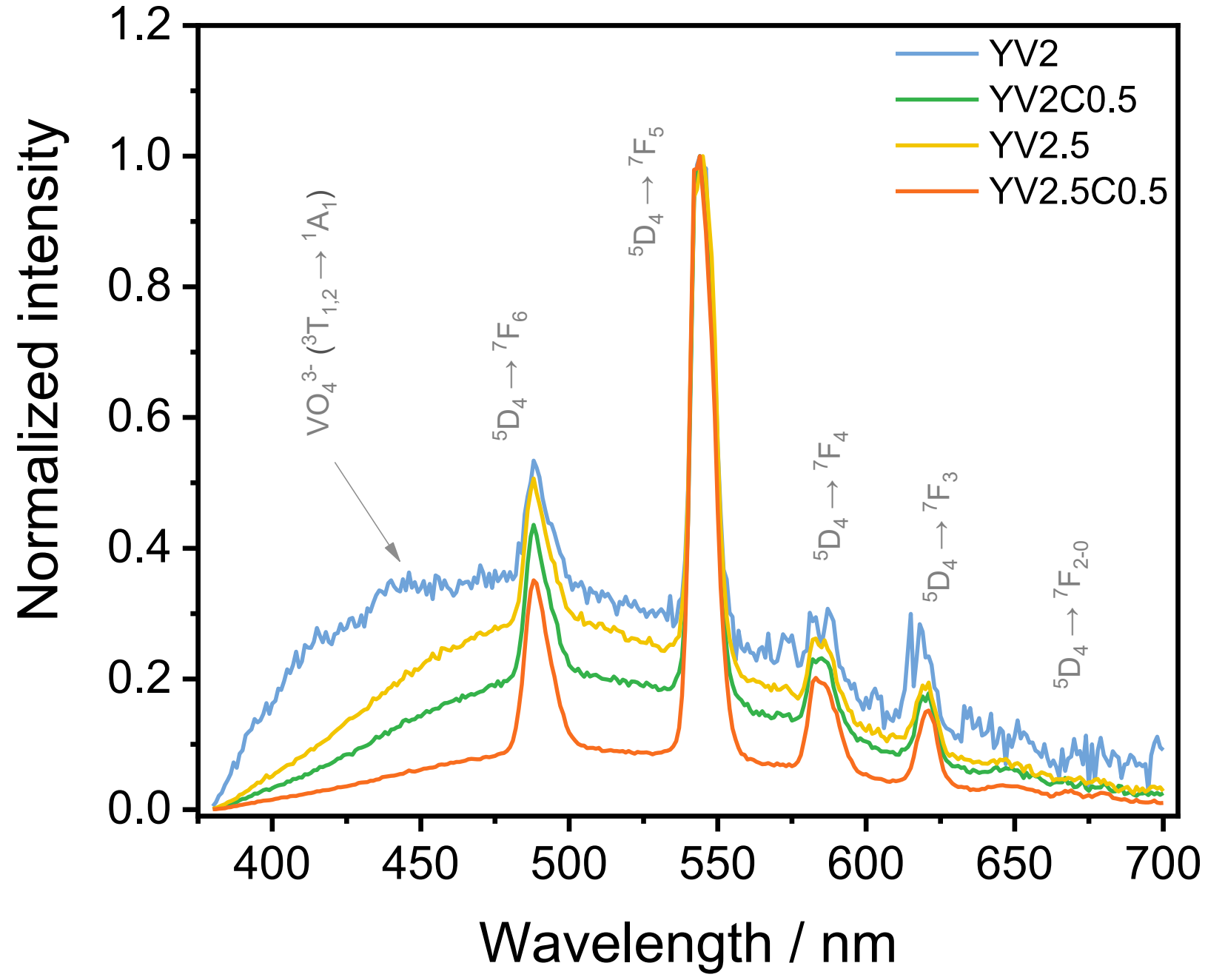

Figure S17. Normalized emission spectra (λ_{exc} = 320 nm) of Tb-doped YV2, YV2C0.5, YV2.5, and YV2.5C0.5 nanoparticles ($(Y_{0.95}Tb_{0.05})VO_4$) measured in powder form. YV*n* and YV*n*C*x* indicate syntheses performed with *n* equivalents of NaOH and *x* equivalents of Na_2CO_3 with respect to the $NaVO_3$ precursor.

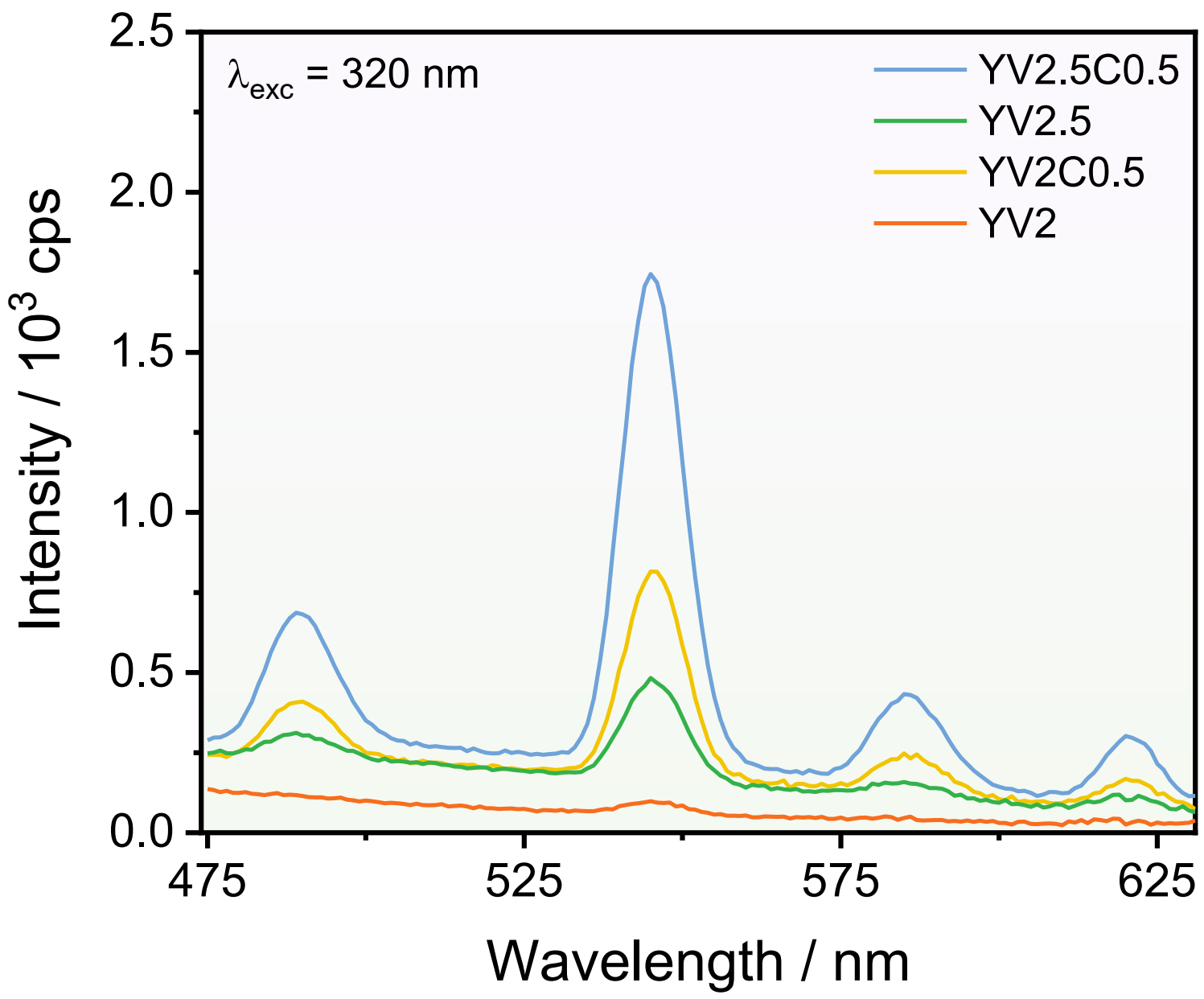

Figure S18. Absolute emission spectra (λ_{exc} = 320 nm) of Tb-doped YV2, YV2C0.5, YV2.5, and YV2.5C0.5 nanoparticles ($(Y_{0.95}Tb_{0.05})VO_4$) measured as powders using an integrating sphere. YV*n* and YV*n*C*x* notations correspond to the syntheses performed with *n* equivalents of NaOH and *x* equivalents of Na_2CO_3 relative to the $NaVO_3$ precursor.

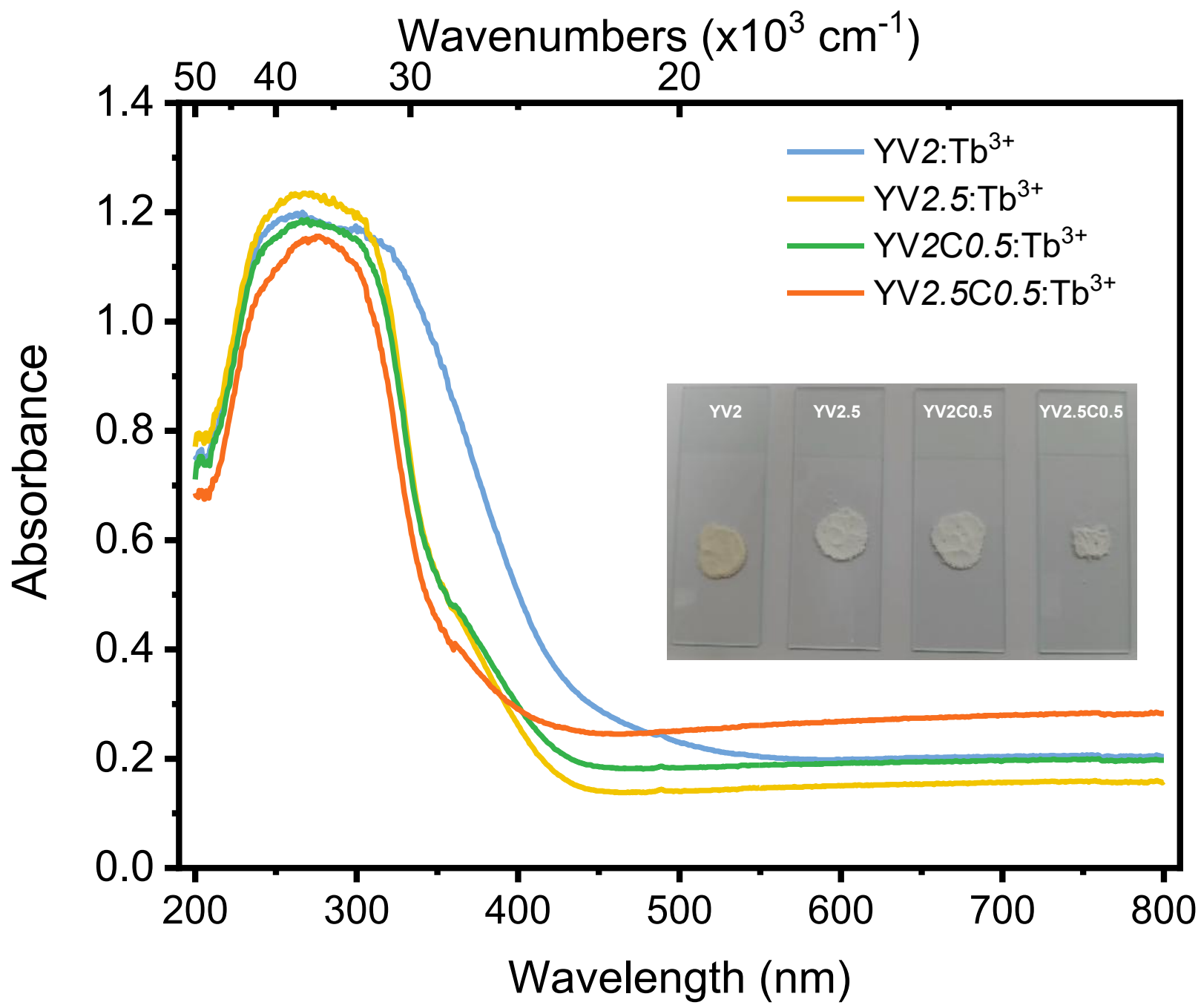

Figure S19. UV-Vis absorption spectra of the Tb-doped YV2, YV2C0.5, YV2.5, and YV2.5C0.5 nanoparticles ($Y_{0.95}Tb_{0.05}VO_4$). The spectra were acquired via diffuse reflectance of powders in a Shimadzu 2550 spectrometer, using barium sulfate as blank. The inset shows photographs of the nanoparticles as powders, highlighting the pale-yellow color of the YV2 sample caused by the MMCT absorption in the visible region, in comparison to the nearly white YV2C0.5, YV2.5, and YV2.5C0.5 samples.

Analysis of Tb^{3+} $^5D_4$ luminescence lifetimes

The experimental $^5D_4$ emission decay curves for the Tb-doped YV2, YV2C0.5, YV2.5, and YV2.5C0.5 samples were well fitted by a tri-exponential decay function (equation S1):

$$I(t) = A_1 e^{(-t/\tau_1)} + A_2 e^{(-t/\tau_2)} + A_3 e^{(-t/\tau_3)} \quad \text{(S1)}$$

where an average lifetime (τ_{av}) was estimated using equation S2:

$$\tau_{av} = \frac{A_1\tau_1^2 + A_2\tau_2^2 + A_3\tau_3^2}{A_1\tau_1 + A_2\tau_2 + A_3\tau_3} \quad \text{(S2)}$$

where τ_1, τ_2, and τ_3 correspond to the individual lifetimes for each type of site, and A_1, A_2, and A_3 are the respective amplitudes.

Table S3. Lifetimes (τ_1, τ_2, and τ_3), pre-exponential factors (A_1, A_2, and A_3), and average luminescence lifetimes (τ_{av}) calculated from fitting of Tb^{3+} luminescence decay curves ($^5D_4$ level, λ_{exc} = 320 nm/λ_{em} = 544 nm) for the Tb-doped YV2, YV2C0.5, YV2.5, and YV2.5C0.5 particles in powder form and dispersions in H_2O and D_2O

Sample	τ_1 / ms	A_1	τ_2 / ms	A_2	τ_3 / ms	A_3	τ_{av} / ms
**Powder**							
YV2	0.033 ± 0.004	0.36 ± 0.02	0.123 ± 0.010	0.42 ± 0.01	0.395 ± 0.009	0.22 ± 0.01	0.235 ± 0.003
YV2C0.5	0.037 ± 0.004	0.30 ± 0.02	0.192 ± 0.018	0.42 ± 0.01	0.709 ± 0.020	0.28 ± 0.02	0.497 ± 0.003
YV2.5	0.035 ± 0.002	0.34 ± 0.01	0.188 ± 0.016	0.39 ± 0.01	0.737 ± 0.021	0.27 ± 0.02	0.527 ± 0.001
YV2.5C0.5	0.058 ± 0.002	0.24 ± 0.01	0.374 ± 0.039	0.33 ± 0.01	1.240 ± 0.027	0.43 ± 0.01	1.034 ± 0.005
**H_2O**							
YV2	0.010 ± 0.001	0.30 ± 0.03	0.073 ± 0.004	0.51 ± 0.03	0.355 ± 0.004	0.19 ± 0.01	0.227 ± 0.001
YV2C0.5	0.020 ± 0.003	0.35 ± 0.02	0.130 ± 0.006	0.40 ± 0.01	0.587 ± 0.009	0.25 ± 0.01	0.423 ± 0.002
YV2.5	0.015 ± 0.001	0.36 ± 0.01	0.114 ± 0.002	0.38 ± 0.01	0.571 ± 0.002	0.26 ± 0.01	0.434 ± 0.003
YV2.5C0.5	0.090 ± 0.038	0.12 ± 0.03	0.531 ± 0.053	0.43 ± 0.02	1.226 ± 0.043	0.45 ± 0.04	0.979 ± 0.002
**D_2O**							
YV2	0.012 ± 0.001	0.42 ± 0.03	0.089 ± 0.005	0.44 ± 0.02	0.534 ± 0.018	0.14 ± 0.01	0.340 ± 0.004
YV2C0.5	0.018 ± 0.004	0.42 ± 0.03	0.152 ± 0.017	0.37 ± 0.02	0.876 ± 0.031	0.21 ± 0.01	0.656 ± 0.010
YV2.5	0.017 ± 0.002	0.49 ± 0.02	0.152 ± 0.010	0.33 ± 0.02	0.977 ± 0.018	0.18 ± 0.01	0.749 ± 0.011
YV2.5C0.5	0.159 ± 0.012	0.18 ± 0.01	0.921 ± 0.044	0.38 ± 0.02	2.661 ± 0.043	0.44 ± 0.02	2.338 ± 0.025
